# SR-BI mediates neutral lipid sorting from LDL to lipid droplets and facilitates their formation

**DOI:** 10.1371/journal.pone.0240659

**Published:** 2020-10-15

**Authors:** Tatyana G. Vishnyakova, Alexander V. Bocharov, Irina N. Baranova, Roger Kurlander, Steven K. Drake, Zhigang Chen, Marcelo Amar, Denis Sviridov, Boris Vaisman, Eugenia Poliakov, Alan T. Remaley, Thomas L. Eggerman, Amy P. Patterson

**Affiliations:** 1 Clinical Center, The National Institutes of Health, Bethesda, Maryland, United States of America; 2 National Heart, Lung and Blood Institute, Bethesda, Maryland, United States of America; 3 National Eye Institute, Bethesda, Maryland, United States of America; 4 National Institute of Diabetes, Digestive and Kidney Diseases, Bethesda, Maryland, United States of America; Fundacao Oswaldo Cruz, BRAZIL

## Abstract

SR-BI binds various lipoproteins, including HDL, LDL as well as VLDL, and mediates selective cholesteryl ester (CE) uptake. HDL derived CE accumulates in cellular lipid droplets (LDs), which also store triacylglycerol (TAG). We hypothesized that SR-BI could significantly facilitate LD formation, in part, by directly transporting LDL derived neutral lipids (NL) such as CE and TAG into LDs without lipolysis and *de novo* lipid synthesis. SR-BI overexpression greatly increased LDL uptake and LD formation in stably transfected HeLa cells (SR-BI-HeLa). LDs isolated from SR-BI-HeLa contained 4- and 7-times more CE and TAG, respectively, than mock-transfected HeLa (Mock-HeLa). In contrast, LDL receptor overexpression in HeLa (LDLr-HeLa) greatly increased LDL uptake, degradation with moderate 1.5- and 2-fold increases of CE and TAG, respectively. Utilizing CE and TAG analogs, BODIPY-TAG (BP-TAG) and BODIPY-CE (BP-CE), for tracking LDL NL, we found that after initial binding of LDL to SR-BI-HeLa, apoB remained at the cell surface, while BP-CE and BP-TAG were sorted and simultaneously transported together to LDs. Both lipids demonstrated limited internalization to lysosomes or endoplasmic reticulum in SR-BI-HeLa. In LDLr-HeLa, NLs demonstrated clear lysosomal sequestration without their sorting to LDs. An inhibition of TAG and CE *de novo* synthesis by 90–95% only reduced TAG and CE LD content by 45–50%, and had little effect on BP-CE and BP-TAG transport to LDs in SR-BI HeLa. Furthermore, intravenous infusion of 1–2 mg of LDL increased liver LDs in normal (WT) but not in SR-BI KO mice. Mice transgenic for human SR-BI demonstrated higher liver LD accumulation than WT mice. Finally, Electro Spray Infusion Mass Spectrometry (ESI-MS) using deuterated d-CE found that LDs accumulated up to 40% of unmodified d-CE LDL. We conclude that SR-BI mediates LDL-induced LD formation *in vitro* and *in vivo*. In addition to cytosolic NL hydrolysis and *de novo* lipid synthesis, this process includes selective sorting and transport of LDL NL to LDs with limited lysosomal NL sequestration and the transport of LDL CE, and TAG directly to LDs independently of *de novo* synthesis.

## Introduction

LDs are cellular organelles that accumulate and store neutral lipids (NL). The LD lipid core contains predominantly CE and TAG surrounded by a phospholipid monolayer and stabilized by structural amphipathic PLIN family proteins [1]. LDs can also reversibly bind various enzymes of lipid metabolism [2, 3]. Mobilization of activated cytosolic neutral lipases, such as hormone sensitive lipase (HSL) and adipose triglyceride lipase (ATGL) to LDs are known to facilitate to LD TAG and CE hydrolysis and release of fatty acids (FA), glycerol and free cholesterol (FC) [4–7].

Classically, LDs are thought to originate in the endoplasmic reticulum (ER) from *de novo* lipid synthesis catalyzed by several key enzymes such as the long chain acyl-CoA synthetase (ACSL1-5), diacylglycerol acyltransferase-1/2 (DGAT-1/2) and acyl coenzyme A cholesterol acyltransferase (ACAT1/2) all located in the ER [2]. Newly synthesized TAG and CE accumulate between the two leaflets of the ER membrane phospholipid bilayer, forming lens-like structures, which bud off the ER membrane forming LDs [1, 2, 8]. LD formation can be stimulated by fatty acids (FA) added to cells *in vitro* or FA derived from lipoprotein TAG hydrolyzed by plasma lipoprotein lipase (LPL) in peripheral tissues [9–12]. FA as well as FC also can be delivered to cells as a result of receptor-mediated lipoprotein lysosomal degradation (lysosomal/acid lipid hydrolysis). For example, the LDL-receptor mediates holoparticle LDL endocytosis to lysosomes and thus facilitates lysosomal hydrolysis of both apolipoprotein B by acid proteases as well as LDL lipids by lysosomal acid lipase (LAL) [13, 14].

In contrast to the LDLr (Low density lipoprotein receptor), SR-BI binds a wider spectrum of lipoproteins including HDL, chylomicrons, VLDL and LDL as well as mediates selective cholesteryl ester uptake [15]. Selective CE uptake is the process whereby CE is internalized while the apolipoprotein containing HDL/lipoprotein-SR-BI complex remains at the plasma membrane or is recycled back after internalization. After uploading some CE to the cell, smaller, CE poor HDL dissociates from the receptor complex [15–17]. Selective CE uptake is the final step in reverse cholesterol transport to the liver and also provides the single most important source of cholesterol in adrenal steroidogenesis [11, 18]. It has been demonstrated that HDL-CE accumulates in the LDs of steroidogenic cells and tissues [19] and does not require CE lysosomal hydrolysis [12, 20, 21]. In addition to CE, HDL-TAG selective uptake has also been reported in SR-BI expressing cells [22]. This suggests that SR-BI could potentially facilitate LD expansion by directly transporting CE and TAG into LDs without sequential cycles of lipid hydrolysis by either neutral cytosolic or acid/lysosomal lipases followed by *de novo* synthesis in the ER. Such a pathway might be a steady-state energy-efficient delivery system for CE and TAG in some tissues. Importantly, steroidogenic tissues including adrenal glands [23], testis [24] and ovary [25], as well as the liver, adipocytes and cells of the reticuloendothelial system (CRES), such as macrophages, accumulate LDs [13, 26–29], and simultaneously express high levels of SR-BI [30, 31]. Unlike the LDLr, whose function is mainly related to LDL/VLDL uptake and subsequent degradation [32], SR-BI binds various lipoproteins mediating selective CE uptake without simultaneous apolipoprotein degradation [18, 33].

Notwithstanding previous reports on the molecular mechanisms of SR-BI function in selective HDL CE uptake, bile excretion and reverse cholesterol transport, the role of SR-BI in lipoprotein dependent hepatic LD formation, accumulation and metabolism is poorly understood. In this study, we assessed the hypothesis that SR-BI mediated lipoprotein uptake leads to selective CE and TAG sorting, internalization, transport and accumulation in LDs. Using human LDL as the primary source of neutral lipids, we demonstrated that LDL loading through SR-BI greatly contributes to increased LD formation *in vitro and in vivo*. We have also found that SR-BI-mediated LDL-CE and TAG transport is not associated with lysosomal degradation, and that CE hydrolysis and *de novo* synthesis were only partially required for NL transport to LDs. Thus, LD formation and expansion can be SR-BI dependent with both direct TAG and CE transport to LDs as well as *de novo* TAG and CE synthesis. Additionally, the role of SR-BI contributing to LD formation was also confirmed *in vivo*, demonstrating the critical importance of SR-BI in liver LD formation.

## Materials and methods

### Materials

BSA (essentially fatty acid free), FASN inhibitor (cerulenin), ACSL-1-5 inhibitor (Triacsin C), ACSL-4 inhibitor (Troglitazone), Diacylglycerol acyltransferase inhibitors (betulinic acid and A922500) and ACAT inhibitor (Sandoz 58–035) were purchased from Sigma-Aldrich (St. Louis, MO). All fluorescent neutral lipid analogs and labeling kits (S1 Fig) were from Invitrogen (Eugene, OR).

#### Mice

SR-BI KO and normal C57BL/6 mice were purchased from Jackson Laboratory (Bar Harbor, ME). SR-BI KO mice were back crossed to C57BL/6N 10 times to create an SR-BI/II KO on a C57BL/6N background. The liver-specific expression vector pLIV.11, which contains the human apo E promoter was used to express SR-BI in the liver. Full-length (1.7-kb) human SR-B1 cDNA (GenBank: BC112037.1) was flanked by Not I linkers and inserted into a unique Not I of modified pLIV.11. Clones with correct orientation of the transgene were selected after digestion of the plasmid DNA by Sph I and Aat II. The obtained pLIV11-hSR-BI plasmid was digested with Sal I and Spe I, and an 11.6-kb DNA fragment LIV-hSR-BI, containing the complete expression cassette was isolated, purified and used for generating the transgenic mice. All animal studies were approved by the Institutional Animal Care and Use Committee of the National Heart Lung and Blood Institute under protocols H-0050R2, H-0050R4 and H-0100R3. Mice are housed in an AAALAC accredited facility in individually ventilated cages with fluffy paper bedding and compressed cotton squares (Nestlets^®^) for enrichment. Water is purified by reverse osmosis then autoclaved. The standard diet is NIH-31. On normal workdays, mice are checked twice daily and once daily on weekends and holidays. Any mice with health issues are reported to the clinical veterinarian for evaluation and treatment. Animals were sacrificed utilizing cervical dislocation, the well-established procedure for minimizing animal stress and suffering. Anti-PLIN1 antibody was from Acris Antibodies, anti-PLIN2 antibody was custom produced in guinea pig by AnaSpec and anti-PLIN3 antibody was purchased from Cell Signaling Technology, Inc.

#### Cell culture

SR-BI-HeLa: HeLa cells were stably transfected with human SR-BI (CLA-1) as previously reported [34]. LDLr-HeLa: HeLa cells were co-transfected with the human LDL receptor and puromycin resistance pcDNA 3.1 plasmids. Puromycin selected HeLa cells demonstrating an increased uptake and internalization of Alexa 488-LDL were selected and LDLr expression was further confirmed by Western blotting and RT PCR. Mock-HeLa: HeLa cells were stably transfected with the puromycin resistance pcDNA 3.1 plasmid and selected with puromycin.

#### Labeling procedures

Human LDL, isolated utilizing the method of Redgrave et al. 1975 [35], were labeled with an Alexa 488 or Alexa 568 protein labeling kit (Invitrogen, Eugene, OR). The Alexa-labeled ligands were analyzed by a 4–20% Tris-Glycine SDS-PAGE, under reducing conditions to confirm covalent labeling. Gels were scanned using a Fluorocsan (model A, Hitachi). Alexa-labeled preparations of lipoproteins migrated at positions expected for their molecular masses. BODIPY-CEs (BP-CE), Cholesteryl BODIPY 542/563 C11 [red] (cat# C12680) and Cholesteryl BODIPY FL C12 [green] (cat#C3927MP) were from Invitrogen, Eugene, OR, BODIPY 542/563 C11 -diacylglycerol (BP-TAG) was synthesized as reported earlier [36]. For lipoprotein labeling, 1 ml of LDL (3–4 mg/ml) was mixed with 1 ml of phospholipid donor micelles (2 mg POPC, 50 μg BP-CE reconstituted in water) and 1 ml of delipidated human plasma and incubated overnight at 37°C. Similarly, BP-TAG donor micelles (2 mg POPC, 50 μg BP-TAG reconstituted in water) were incubated with LDL but without human plasma overnight. Labeled LDL were re-isolated by affinity chromatography, using the Seppro^®^ LDL removal kit (GenWay, USA). For obtaining double labeled particles, LDL was first labeled with BP-CE, re-isolated and labeled with BP-TAG and re-isolated again. The chemical formulas and LDL labeling strategy are shown in S1 and S2 Figs. To prevent potential oxidation, the labeling mixtures were always incubated under N_2_. In contrast to minimally oxidized LDL which induced a 2-4-fold an increase in IL-8 secretion in THP-1 cells, BP-CE, BP-TAG and dual BP/CE/TAG labeled LDL did not induce IL-8 secretion in THP-1 cells indicating that BP-NL labeled LDL were not or were less than minimally oxidized (data not shown). Labeling of LDL with ^125^I-Na was performed by the N-bromosuccinimide method according to Sinn et al. [37]. The specific radioactivity ranged from 1000 to 3000 cpm/ng of protein, with more than 98% of the radioactivity being protein associated.

#### Lipoprotein uptake and degradation

Lipoprotein uptake was measured in DMEM containing 2 mg/ml BSA and 20 mM HEPES. Confluent mock-, hSR-BI- and LDLr-HeLa were incubated with various concentrations of either Alexa488-LDL or ^125^I-labeled LDL at 37°C for 2 hrs, then washed extensively with ice cold phosphate-buffered saline (PBS), and detached with Cell stripper dissociation solution (Cellgro, Herndon, VA). LDL degradation was analyzed as previously reported [34]. Briefly, various cells were incubated with 5 μg/ml of ^125^I- LDL in the presence or absence of 40-fold excess of unlabeled LDL for various times. Conditioned media was collected, and LDL precipitated with 10% TCA acid. Non-precipitated radioactivity in media was counted and considered as degraded LDL. Specific degradation was calculated as the difference between total (in the absence of cold LDL) and nonspecific (in the presence of 40-fold excess of cold LDL) counts.

#### Immunofluorescent and confocal microscopy

HeLa cells were grown on glass chamber slides (Nagle Nunc, Rochester, NY) until 20–50% confluence, rinsed with PBS and further incubated in serum-free DMEM for 24 h at 37°C to eliminate endogenous LDs. 10 μg/ml of fluorophore labeled LDL (Alexa 488/568, protein labeled LDL, LDL-BODIPY-CE, LDL- BODIPY-TAG or combinations) were added to cells for 3 hr. at 37°C. Cells were washed and further incubated with Alexa 488 labeled transferrin (30 minutes), 0.5 μM of neutral BODIPY (1 minute) or Lysotracker Red DND-99 or LysoTracker^™^ Green DND-26 (30 minutes). After nuclei staining with Hoechst 33342 (1 μg/ml), cells were rinsed with PBS and analyzed live using a Zeiss 780 confocal system (Zeiss, Jena, Germany). For immunofluorescent staining, cells were fixed in 3.7% paraformaldehyde in PBS and processed as previously reported [34]. Images were acquired sequentially by using a 488-nm laser line and emission between 505 and 580 nm for Alexa Fluor 488, BODIPY 493/503 and BODIPY FL C12. A 594-nm laser line and emission >610 nm was used for Alexa 594 and a 561-nm laser line and emission between 575–615 nm was used for Alexa 568 and BODIPY 542/563 C11. Z-series high-resolution (100 nm per pixel) images were obtained with a 63x, 1.4-numerical-aperture Plan-Apochromat oil-immersion objective under conditions avoiding bleed-through. In addition to visual imaging, co-localization was quantified using threshold-based analyses (Imaris software, Bitplane, Zurich). Co-localization was assessed and pixel co-distribution was calculated for green and red staining patterns throughout the 3D data sets. The percentage (%) of co-localized fluorescent labels (by expressing intensity multiplied by volume) was compared. Statistics including a Pearson’s Correlation for whole images was calculated.

#### LD isolation, TAG and cholesterol measurements and thin layer chromatography

LDs were isolated as previously described [9] with small modifications. HeLa cells expressing various lipoprotein receptors were incubated with 200 μg/ml of human LDL or without lipoproteins in serum free DMEM overnight. Medium was removed, and cells were rinsed twice with ice-cold phosphate-buffered saline (PBS) before scraping cells into PBS. Cells from each 150 mm Petri dish (containing average 20 ±2x10^6^ cells) were pelleted by centrifugation at 500 *g* for 5 min. Cell pellets were re-suspended in 2 ml of hypotonic lysis solution (HLS) containing 10 mM Tris, pH 7.4, 1 mM EDTA, 10 mM sodium fluoride, 20 μg/ml leupeptin, 1 mM benzamidine, and 100 μM 4-(2-aminoethyl) benzenesulfonylfluoride hydro-chloride by pipetting and incubated on ice for 10 min before homogenization by 10 strokes in a Teflon-glass homogenizer. A post-nuclear fraction was collected after centrifugation at 1,000 *g* for 10 min. 2 ml of post-nuclear supernatant were added to 1 ml of 60% sucrose stock solution in HLS, layered with 5 ml of 5% sucrose solution in HLS and 2 ml of HLS. Gradients were centrifuged at 30000 RPM at 4°C in a SW41Ti rotor (Beckman) for 30 min. The floating LD layers were harvested using a Beckman tube slicer, reconstituted with 5% sucrose in HLS and centrifuged again. LD lipids were extracted utilizing the Folch procedure. In some experiments, LDs were further dialyzed against water, lyophilized and used for western blotting. TLC was performed on Silica gel G 60 10 × 10 cm (Merck) using a mixture of hexane/diethyl ether/formic acid (80:20:2 by vol.). Lipids on TLC plates were developed by a Molybdenum Blue reagent (Sigma, # M1942) reagent followed by heating at 160°C for 10–15 min and scanned with a densitometer operated in the reflectance mode (GE Healthcare). The relative density of TAG, CE and CH bands were measured utilizing an ImageJ program (NIH, Bethesda). The non-polar lipid mixture (Matreya # 1130) was used as a standard. Data was presented as arbitrary units for each lipid in LD preparations. TAG, and TC were also measured in LDs from samples with equal protein content utilizing a fluorometric assay kit (BioVision, # K614 and # K603). For DESI-MS, LD preparations were extracted using the Folch method, extracted lipids were subjected to TLC (conditions above) and TAG and CE were extracted from bands cut from silica plates with chloroform.

#### MS analysis (tryptic digestion)

Isolated LDs were mixed with SDS-PAGE sample buffer and boiled for 4 minutes under reducing conditions. Samples were subjected to a 4–10% SDS-PAGE. Coomassie-stained bands were excised from the gel. Destaining, reduction, alkylation, and tryptic digestion were accomplished on a Discover System (CEM Corporation, Matthews, NC), according to the manufacturer’s instructions. The resulting peptides were recovered and concentrated over C18 hydrophobic spin columns (Vivascience, Hannover, Germany), according to the manufacturer’s instructions.

#### Mass spectrometry analysis (MALDI acquisition and analysis)

Recovered trypsin digested peptides were mixed with saturated a-cyano-4-hydroxycinnamic acid and analyzed in positive ion mode by MS and MS/MS using a MALDI AutoFlex III TOF/TOF (Bruker Daltonics, Billerica, MA). External calibration with Peptide Calibration I Standards (Bruker Daltonics) encompassed the range of the peptides sequenced. Peaks in both the peptide map spectra and peptide sequencing spectra were evaluated manually and the combined spectra peak lists were submitted to Mascot (Matrix Science, Boston, MA) *via* the Bruker Daltonics data analysis programs FlexAnalysis (v3.0) and BioTools (v3.2). The NCBInr database (11/19/09 version) was searched allowing for carbamidomethylation, oxidation (MHW), and N-terminal acetylation and formylation. Acceptable identity was defined by a Mascot score indicating a p<0.05.

#### LD staining

Tissue neutral lipid accumulation was assessed in mouse livers as described previously (49). Briefly, mice received 2 mg of human LDL intravenously using a retro-orbital injection (IV). The mice were euthanized 2 hours later for collecting blood and organs. In separate experiments, SR-BI KO mice received IV 2x10^8^ PFU adenovirus V5 expressing hSR-BI or Luciferase. At day 3, mice were IV administered 2 mg of human LDL and 2-hs later were euthanized to collect blood and organs. Livers were washed with 10 ml of PBS through the portal vein before freezing on dry ice. 8–12 μm liver and adrenal frozen sections were stained for LDs utilizing 0.5 μM neutral BODIPY in PBS for 5 minutes, washed with PBS, and analyzed by confocal microscopy. *In vitro*, various stably transfected HeLa cells were incubated with 200 μg/ml of LDL for 6 and 18 hours and stained live for LDs as described for organ sections.

### Synthesis of deuterated CE and TAG

Labeled cholesteryl esters (CE) and triacylglycerides were synthesized as previously described with small modifications [36]. Briefly, 1 mmol of fatty acid (Oleic acid-9,10-d2) (284.47 mg/mmol) and 2.6 mmol (393.63 mg/mmol) of Cholesterol-25,26,26,26,27,27,27-d7 or palmitic acid-1-13C (257.42 mg/mmol) and 1,3-di-(9Z-octadecenoyl)-2-hydroxy-sn-glycerol-d5 (DAG) (2.6 mmol) to which 1.2 mmol 4-dimethyl-aminopyridine (146.60 mg) was added. Reagents were dissolved and well mixed in 5 ml of dry methylene chloride. Separately 1.6 mmol N, N-dicyclohexyl carbodiimide (1.6 mole, 330.12 mg) was dissolved in 5 ml dry methylene chloride. The two solutions were mixed and incubated at room temperature overnight. Reactions were performed in a sealed ampule flushed with argon. Products were separated on preparative HPLC as reported earlier [36].

### Lipid standard and sample preparation

Equimolar lipid standard mixture stock solutions were prepared by dissolving TAG and CE lipid standards in chloroform with the concentration of each lipid being 1 M. Working solutions/samples were prepared by diluting the standard stock solution or extracted from TLC plate containing TAG or CE using chloroform, with the addition of an appropriate volume of 1 mM additive stock solution of NaI, dissolved in methanol. For the linearity test experiments, the solutions were 1–200 nM equimolar lipid standard mixtures dissolved in a mixture of chloroform.

### Electro spray infusion mass spectrometry

A quadrupole-time-of-flight mass spectrometer (Q-TOF II; Waters Corp.) was used for the MS analysis with no isolation applied. The quadrupole worked as an ion guide and was operated in RF only mode, where the ion transmission was controlled by ramping the mass 1 of m/z 2 to mass 2 of m/z 700 with no dwelling on either of the two masses. According to the manufacturer, ‘‘for a given quad mass, there is no transmission below 80% of the mass. The transmission decays much more slowly on the opposite side of the mass scale, with transmission still good at three times the set mass.” This MS profile for ion transmission worked well for sensitive detection and reliable quantitation of lipids. The samples were directly infused into the mass spectrometer at a flow rate of 10 to 40 L/min. The spectra were acquired in the 100 to 2000 m/z range. Cone voltage was set at 15 or 50 V unless otherwise indicated. The source temperature was set at 100°C and the desolation temperature was typically set at 150°C or 250°C unless otherwise indicated. Capillary voltage was 3.2 kV; desolvation gas flow rate was 500 L/h. The instrument was externally calibrated with cluster ions of NaI as recommended by the manufacturer [38]. The spectra were smoothed twice using the Savitsky-Golay method and centered based on the peak area or peak height. No background subtraction was performed as it affected quantitation reliability. The deposition of involatile NaI ions on the sample cone was minimized due to the Z-spray design of the Q-TOF II instrument (Waters Corp.); the low concentration applied (typically 100 M); and moving the probe away from the sample cone. To minimize impurity peaks, the syringes and ESI capillary were thoroughly rinsed or flushed multiple times, using a clean solvent or solvent mixture (methanol, chloroform, or methanol/chloroform mixture) in a vial or test tube containing no leftover from last washes for each subsequent wash. The absence of impurities in this setup was verified by direct infusion MS analysis of the solvent mixture before the analysis of the samples.

## Results

### Lipoprotein uptake in HeLa cells stably transfected with hSR-BI and the hLDLr

LDL is metabolized mainly through the LDLr, which mediates both LDL internalization and degradation [39]. While LDLr does not bind HDL, SR-BI mediates both LDL and HDL uptake without apparent apolipoprotein degradation [40]. To assess SR-BI/LDLr functions in receptor mediated LDL metabolism, we utilized hSR-BI-HeLa and hLDLr-HeLa. As a measure of receptor function, ^125^I-LDL uptake and degradation was first compared to mock-transfected HeLa cells which have low endogenous levels of both receptors.

**Fig 1:**
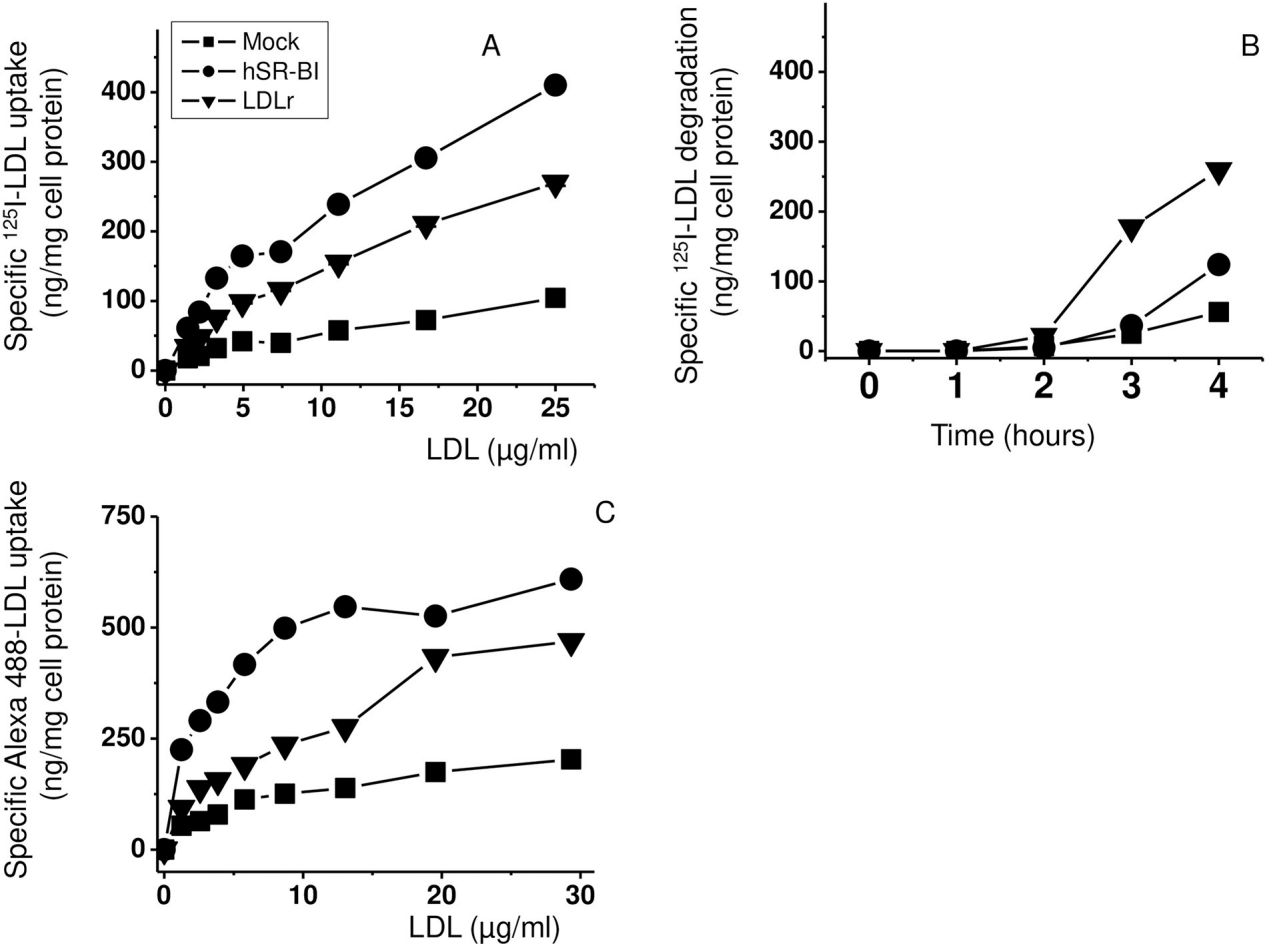
LDL uptake and degradation in HeLa cells. Mock transfected, hSR-BI overexpressing and LDLr overexpressing HeLa cells were incubated with various concentrations of ^125^I labeled LDL (A) and Alexa 488-labeled LDL (C) for 2 h at 37 C° (A, C) or with 5 μg/ml of ^125^I-LDL in the absence or presence of 20-fold excess of unlabeled LDL while collecting media and measuring LDL degradation at 0, 1, 2 and hours (B). The specific dose-response/time course data points represent the difference in radioactive counts measured in the presence and absence of 20-fold excess of unlabeled LDL. In all cases (Panels A, B and C), the differences in both specific LDL uptakes and LDL degradation were statistically significant at 3 hours with p<0.001 between all three groups: hSR-BI-HeLa versus LDLr-HeLa or versus Mock-HeLa.

Specific uptake analyzed at 37°C as a difference between cellular radioactive counts in the presence or absence of 20-fold excess cold ligand. In a range between 1 and 25 μg/ml of LDL, specific uptake was 3- and 4-times higher in LDLr-HeLa and hSR-BI-HeLa, respectively, than in Mock-HeLa (Fig 1A and 1C). While ^125^I-LDL uptake was statistically higher by 25% in hSR-BI-HeLa than in LDLr-HeLa, its degradation (TCA soluble media count, see “Materials and methods”) was time dependent and increased 3-5-fold in LDLr-HeLa when compared to both hSR-BI-HeLa and Mock-HeLa (Fig 1B). In addition to ^125^I-LDL, fluorophore-protein labeled LDL could be utilized for analyzing ligand/receptor interactions and lipoprotein trafficking. As seen in Fig 1C, Alexa 488- protein labeled LDL uptake was similar to ^125^I-LDL uptake indicating that Alexa 488-LDL is an alternative to iodine labeled lipoproteins.

#### LDL-mediated lipid accumulation in HeLa cells

**Fig 2:**
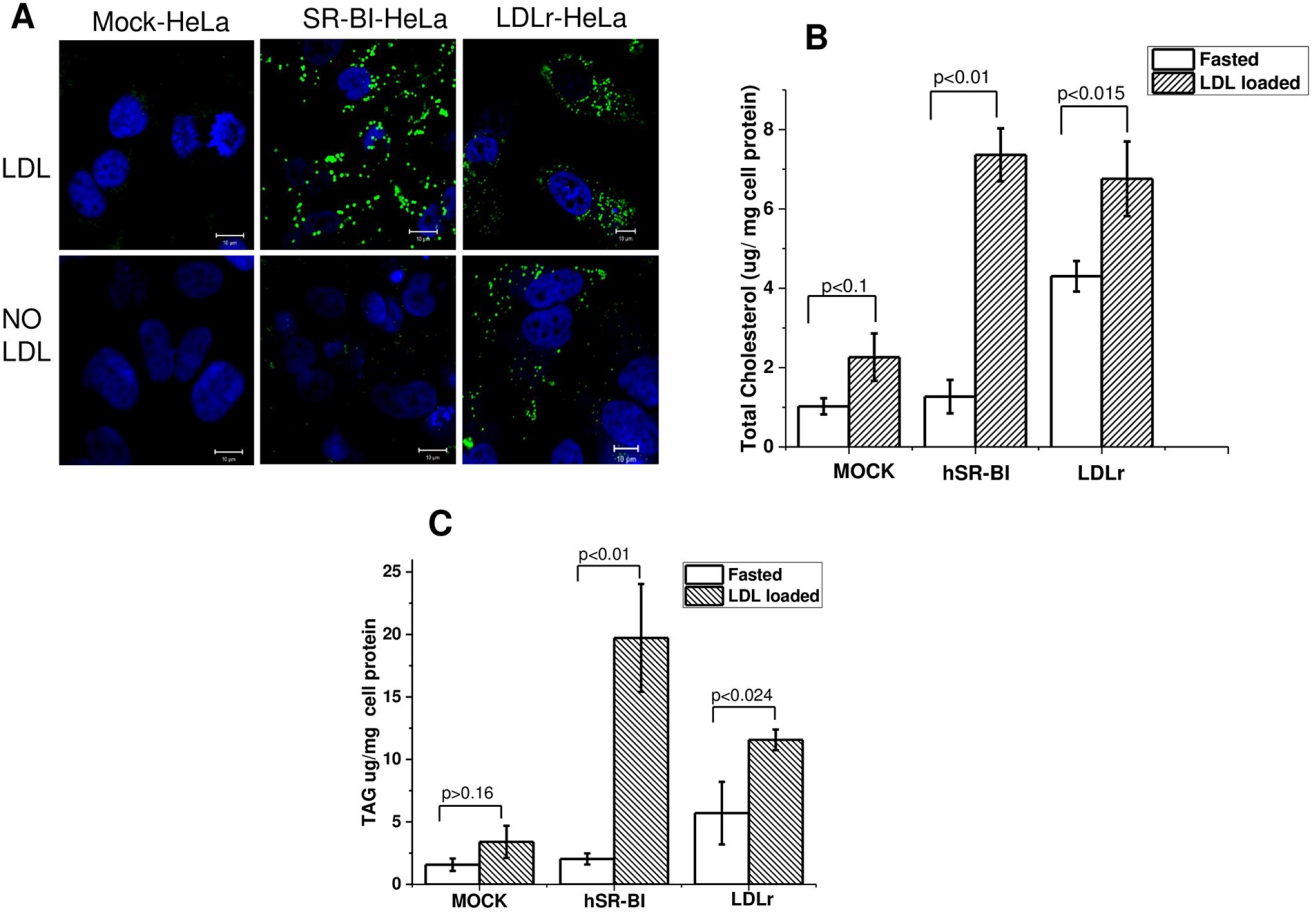
Effect of hSR-BI and LDL receptors on LD accumulation in HeLa cells. hSR-BI and hLDLr stably transfected HeLa cell lines were incubated for 6 hours in the presence or absence of 200 μg/ml of LDL and stained live with 0.5 μM of neutral BP for 1 minute. The cells were then washed, and LDs were immediately visualized utilizing confocal microscopy. Z-stack images are presented for all cells (panel A). The cells were also used for LD isolation as described in Materials and methods. Total cholesterol (panel B), and TAG (panel C) were quantified. Mean ± standard deviation (SD) is shown. The bar corresponds to 10 μm.

LDs are a neutral lipid storage compartment, which accumulate both CE and TAG. Since SR-BI and LDLr mediate LDL uptake and could represent two alternative mechanisms contributing to LD formation [41], we investigated whether these receptors mediate NL accumulation and induce LD formation in HeLa cells. After a 24 h incubation in lipid-free DMEM to reduce background levels of LDs, the HeLa cells were loaded with 200 μg/ml of LDL for 6–18 hours. After a 6-hour incubation, hSR-BI HeLa exhibited a remarkable increase in LD staining when compared to LDL loaded mock-HeLa (Fig 2A). Interestingly, hLDLr-HeLa demonstrated a significant background even after 48 hours of incubation in lipid-free DMEM; however, additional LD accumulation was much smaller in LDL-loaded LDLr-HeLa compared to SR-BI-HeLa.

As seen in Fig 2B and 2C and S3 Fig, LDs accumulated almost 3-times more TAG than TC, and the accumulation of both was increased by 7-10-fold and 2-3-fold in hSR-BI- and LDLr-HeLa, respectively, when compared to Mock-HeLa and the quality of LD isolation was confirmed by MS analysis and western blotting (S4 and S5 Figs).

#### TAG and CE sorting during receptor-dependent LDL uptake

HeLa cells have relatively low endogenous levels of SR-BI and LDLr, and demonstrate a small LD accumulation following LDL loading (Fig 2). hSR-BI- and hLDLr-HeLa with their high lipoprotein uptake and LD formation provide useful models for studying trafficking of lipoprotein and their lipids *in vitro*.

**Fig 3:**
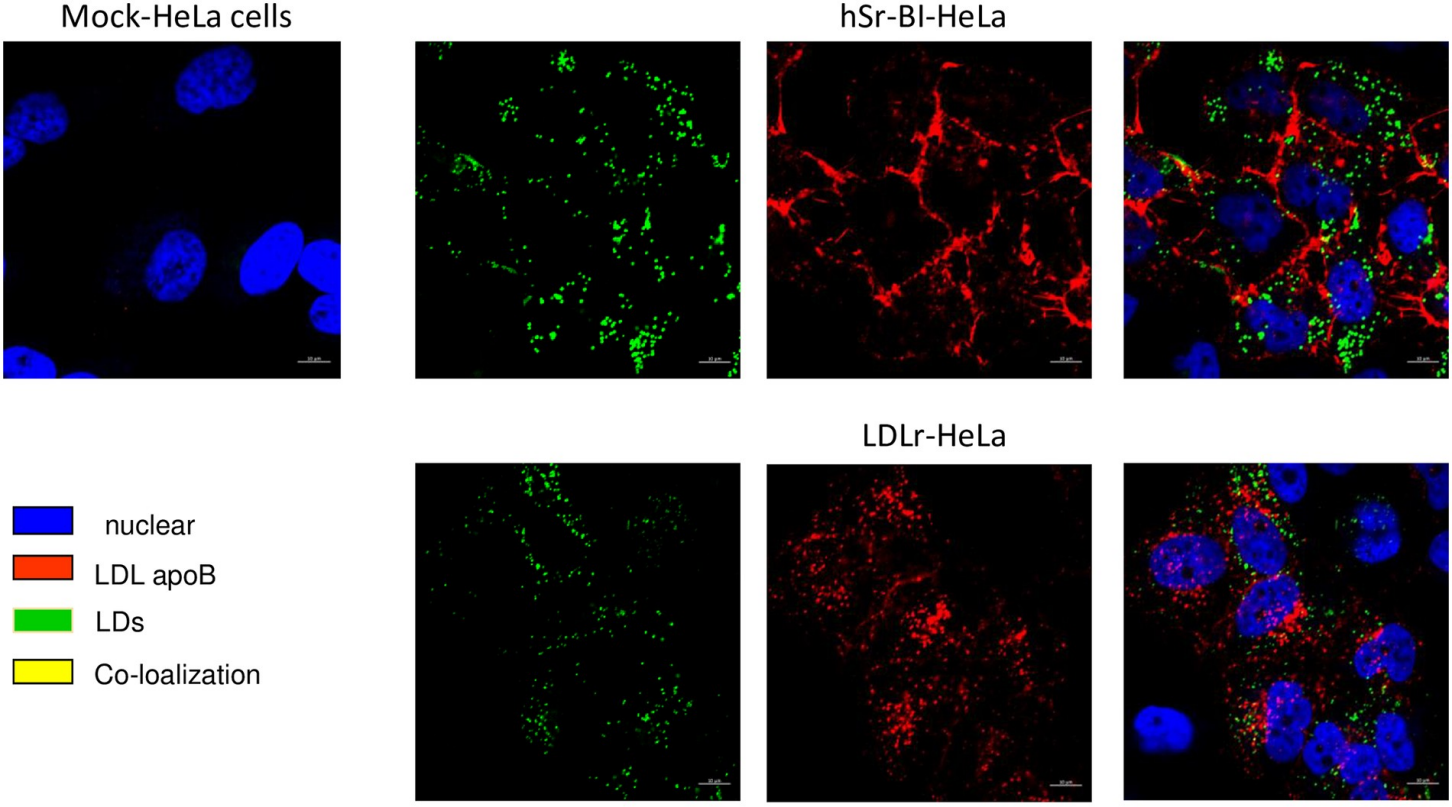
The confocal images of protein labeled LDL in various HeLa cell preparations co-localized with neutral BP-stained LDs. HeLa cells were plated at 30% confluency on glass covered slips and then pre-incubated in serum free media for 24 hours prior to experiments. Labelled LDL were added to the media for 1 hour, washed and chased for 30–60 minutes in serum/lipoprotein free media and stained live with 0.5 μM of neutral BP for 1 minute. The bar corresponds to 10 μm.

LDL cellular lipid transport was first assessed by analyzing the co-localization of protein-labeled LDL (S1 and S2 Figs) with cytosolic LDs. As seen in Fig 3, after 1 hour of incubation most Alexa 568-LDL (Alexa 568-covalently labelled LDL apoB, red) was present predominantly at the cell surface, with only a small amount of internalization in SR-BI HeLa. Additionally, co-localization of the internalized Alexa 568-LDL with LDs (neutral BODIPY, green) was very minor and limited to sites where LDs resided closely to the cell surface of hSR-BI-HeLa. Alexa 568-LDL was robustly internalized in LDLr-HeLa, but demonstrated no co-localization with LDs.

**Fig 4:**
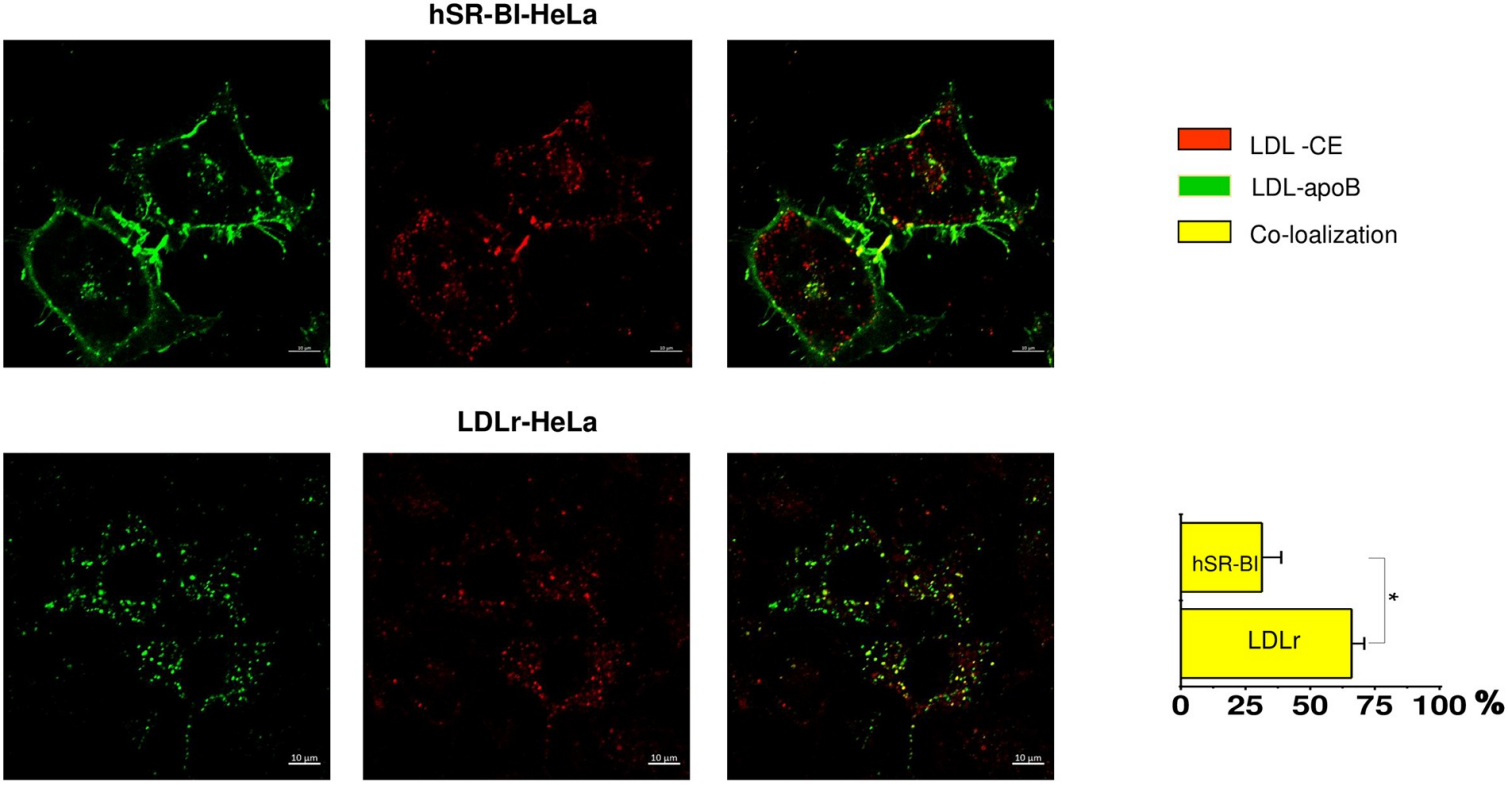
Dual -CE/protein-labeled LDL uptake and sorting. HeLa cells were plated at 30% confluency on glass covered slips and then pre-incubated in serum free media for 24 hours prior to experiments. CE/protein-labeled LDL were added to the media for 1 hour, washed and chased for 30–60 minutes in serum/lipoprotein free media. The data is arranged in rows with a color code seen on the right side of the panels. The bar corresponds to 10 μm, *p < 0.015, **p<0.01.

**Fig 5:**
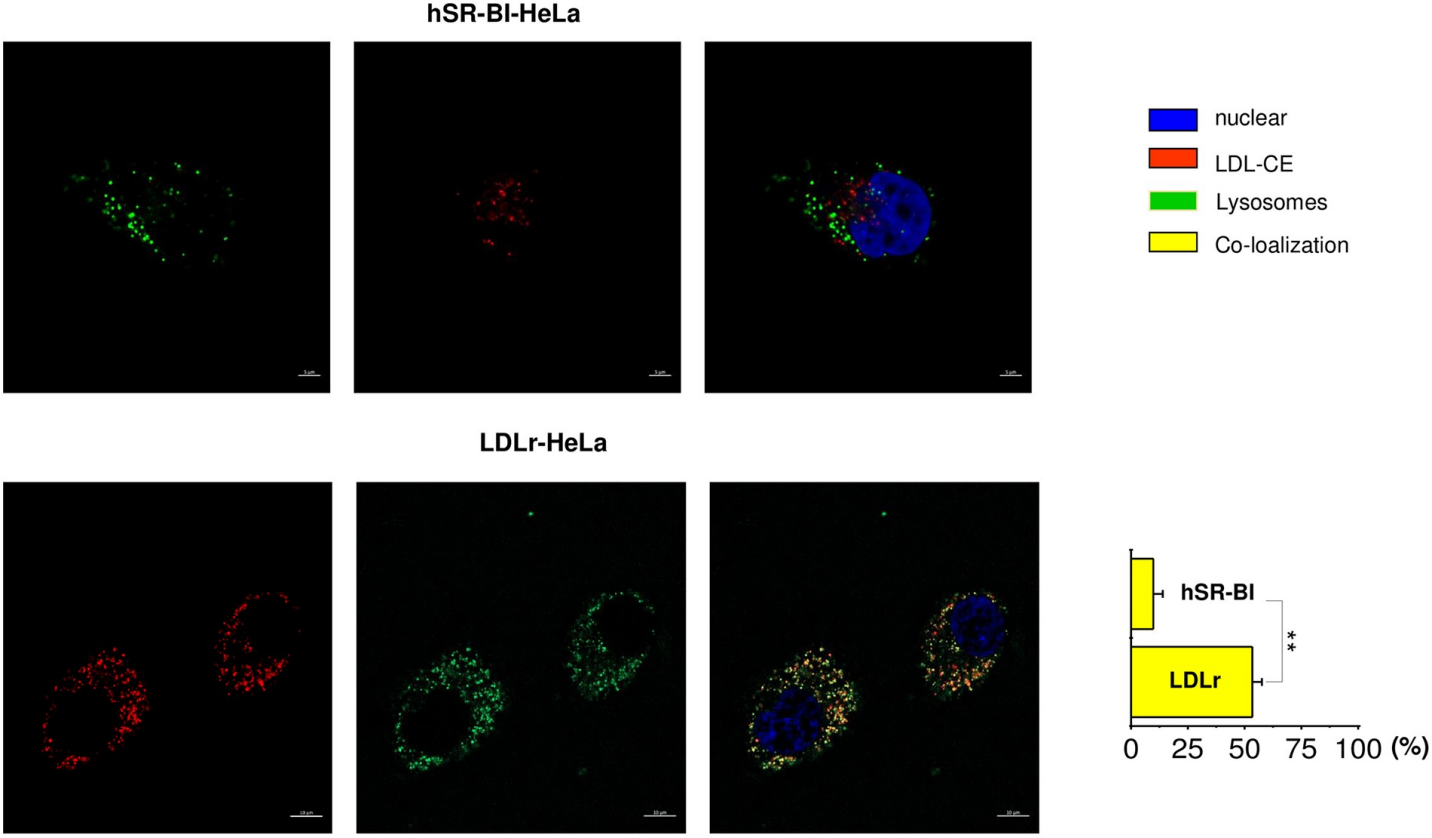
Transport of CE-LDL to the lysosomal compartment in various HeLa cells. HeLa cells were plated at 30% confluency on glass covered slips and then pre-incubated in serum free media for 24 hours prior to experiments. Labelled CE-LDL were added to the media for 1 hour, washed and chased for 30–60 minutes in serum/lipoprotein free media. In the presence of Lysotracker Green (lysosomal marker). The data is arranged in rows with a color code seen on the right side of the panels. The confocal images of CE labeled LDL internalization to lysosomes utilizing Lysotracker green are shown for mock, SR-BI and LDLr HeLa cells as indicated at the top of the panels. The bar corresponds to 10 μm, * p < 0.015, ** p<0.01.

Because apoB-labeled LDL was not directly transported to LDs, we further assessed whether and where CE is sorted from apoB-labeled LDL holoparticles in SR-BI-HeLa. When cells were incubated for 1 hour with dual, apoB protein(green)/BP-CE (red) labeled LDL following a 2-hour chase incubation (Fig 4), Alexa 488-ApoB labeled LDL remained mostly at the cell surface while BP-CE was internalized in SR-BI-HeLa. In contrast, LDLr HeLa rapidly internalized LDL as a holoparticle with Alexa488-apoB and BP-CE remaining co-localized (Fig 4). When using the proportion (%) of BP-CE co-localized with Alexa-apoB as a measure of holoparticle versus selective LDL NL uptake, only a relatively small amount (27.2±10.6%) of apoB and BP-CE remained co-localized (yellow) at the cell surface in SR-BI-HeLa In contrast, LDLr-HeLa exhibited a much higher co-localization of internalized apoB and BP-CE (63.5%±9.6), which was consistent with LDL holoparticle uptake. Because the LDLr is known to mediate LDL endocytosis to lysosomes, a site of acid lipid hydrolysis [5], we next compared the extent of LDL BP-CE lysosomal endocytosis in SR-BI- versus LDLr-HeLa cells. Using Lysotracker Green (green), a fluorescent dye that preferentially accumulates in late endosomes and lysosomes (LS), the % of BP-CE (red) co-localized with LS was calculated in HeLa cells. Only a minor fraction (9.2±2.3%) of internalized BP-CE was co-localized with Lysotracker (yellow) in SR-BI-HeLa. A much higher amount, 52±7.1% of BP-CE, was located with the LS of LDLr-HeLa (Fig 5 lower panels).

#### LDL NL transport to LDs

**Fig 6:**
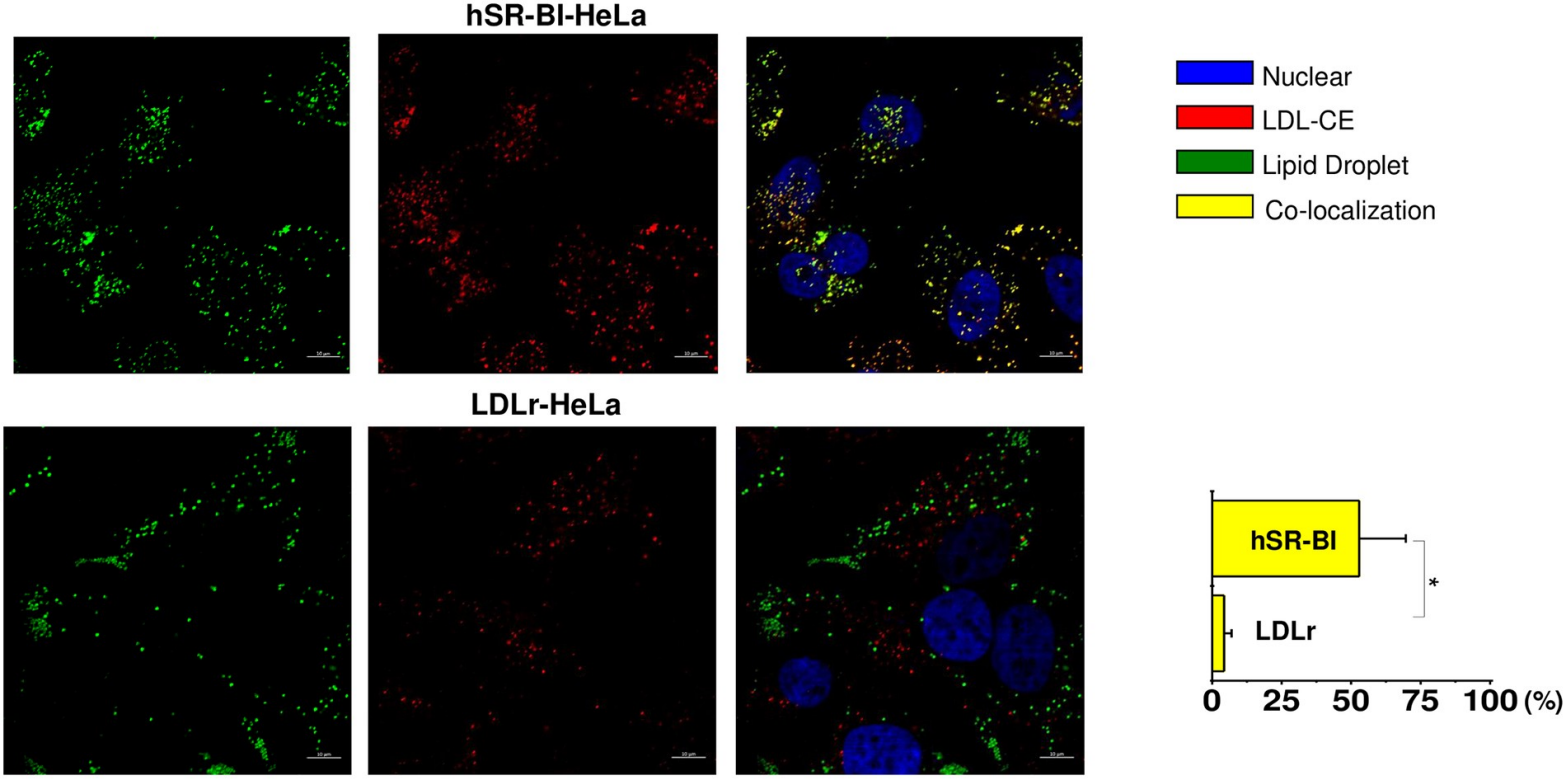
Co-localization of CE labeled LDL with neutral -stained LDs. HeLa cells were plated at 30% confluency on a glass cover slips and pre-incubated in serum free media for 24 hours prior to experiments. Labelled LDL were added to the media for 1 hour, washed and chased for 60 minutes in serum/lipoprotein free media. Cells were stained for LDs utilizing Neutral BODIPY as described in Materials and methods. The confocal images of -CE labeled LDL co-localized with neutral -stained LDs are demonstrated for various receptor expressing HeLa cells. The color code and extent of co-localization are shown in the right columns. The cell type is specified at the top of the figure. The bar corresponds to 10 μm, * p < 0.001.

We next studied whether both CE and TAG from LDL are internalized to LDs facilitating LD formation and expansion in a SR-BI-dependent manner. Using BP-CE, BP-TAG and dual, BP-CE/BP-TAG labeled LDL, we assessed the percent of LDL CE and TAG co-localization with LDs stained with Neutral BODIPY. After a 2-hour pulse/chase incubation with BP-CE LDL (red), about 51.5±5.2% of LDL CE was sorted to LDs (green) in SR-BI-HeLa (Fig 6, upper panels). A smaller (2.5±1.5%) amount of LDL BP-CE was co-localized with LDs in LDLr-HeLa.

**Fig 7:**
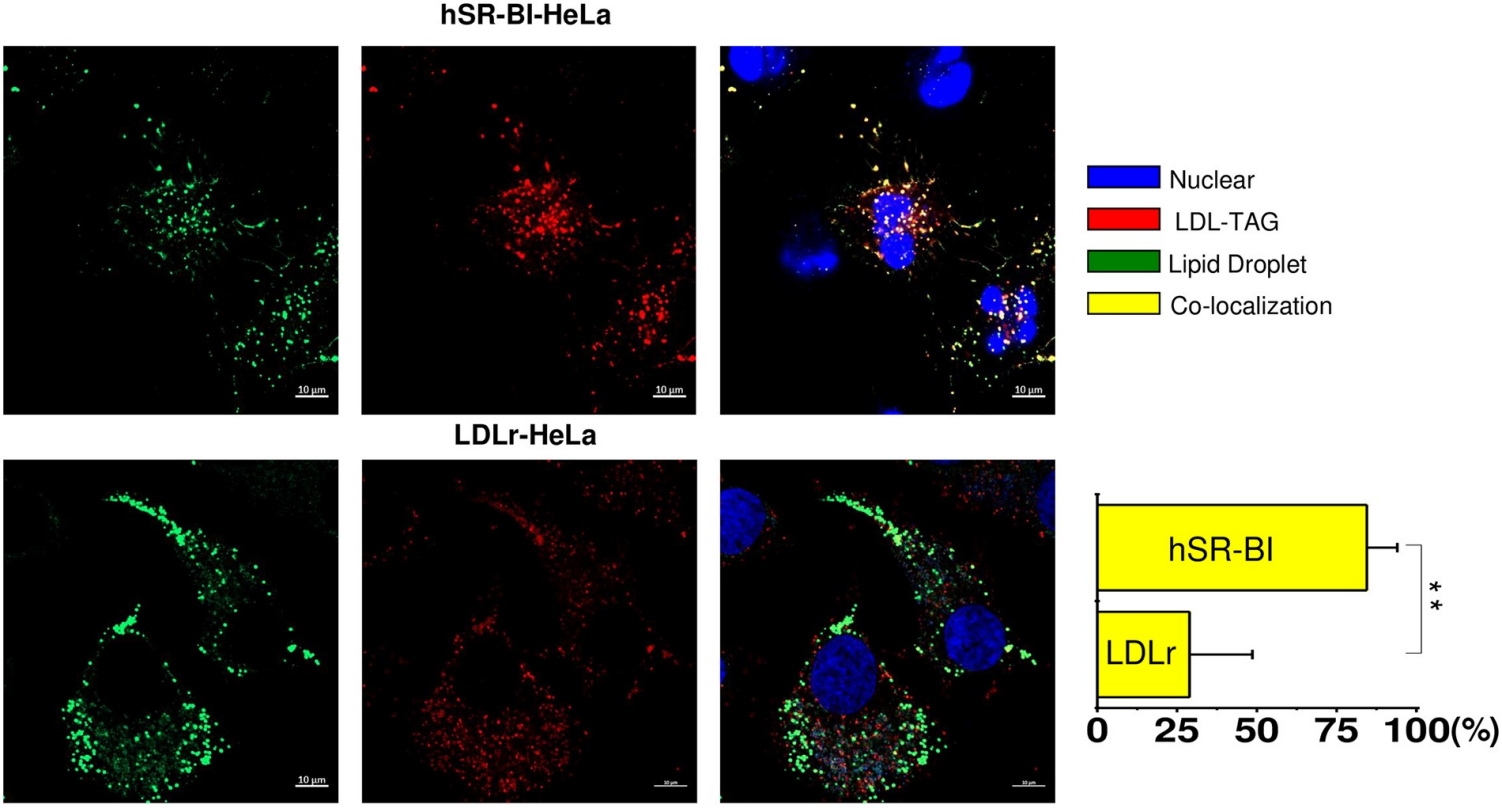
Co-localization of TAG labeled LDL with neutral BP -stained LDs. HeLa cells were plated at 30% confluency on glass cover slips and pre-incubated in serum free media for 24 hours prior to experiments. Labelled LDL were added to the media for 1 hour, washed and chased for 60 minutes in serum/lipoprotein free media. Cells were stained for LDs utilizing Neutral BODIPY as described in Materials and methods. The confocal images of -TAG labeled LDL co-localized with neutral BP-stained LDs are demonstrated for various receptor expressing HeLa cells. The color code and extent of co-localization are shown in the right columns. The cell type is specified at the top of the figure. The bar corresponds to 10 μm, ** p < 0.01.

Co-localization of BP-TAG (red) labeled LDL with LDs (green) in SR-BI-HeLa and LDLr HeLa cells is seen in Fig 7 LDL BP-TAG was internalized and sorted to LDs with 85±12.8% of the TAG co-localized with LDs (yellow). The co-localization was much smaller in LDLr-HeLa (26.3±12.1%).

**Fig 8:**
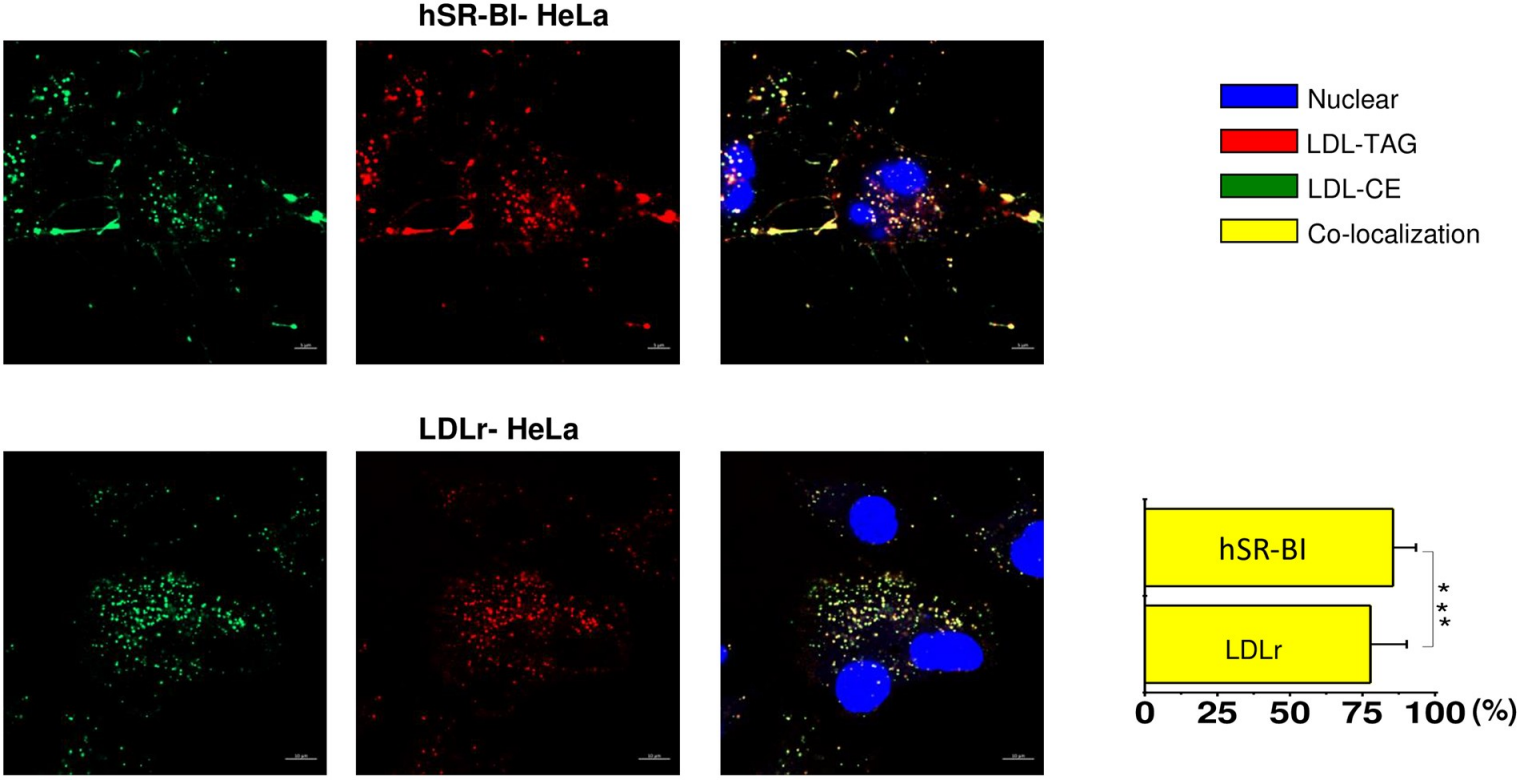
Transport of LDL core NL in various HeLa cells. HeLa cells were plated at 30% confluency on a glass cover slips and pre-incubated in serum free media for 24 hours prior to experiments. Dual BP-CE/BP-TG labeled LDL were added to the media for 1 hour, washed and chased for 60 minutes in serum/lipoprotein free media. The color code and extent of co-localization are shown in the right columns. The cell type is specified at the top of the figure. The bar corresponds to 10 μm, *** p>0.1.

Finally, since TAG and CE are NL residing within a lipoprotein core, we studied whether CE and TAG are transported together utilizing BP-CE and BP-TAG dual labeled LDL. As seen in Fig 8 (upper panels), CE and TAG were found mostly co-localized in both SR-BI HeLa (86±12.9%), and in LDLr-HeLa cells (77.4±12.3%), indicating that LDL core NL were transported simultaneously and together.

#### Role of de novo neutral lipid synthesis in LD formation by SR-BI

**Fig 9:**
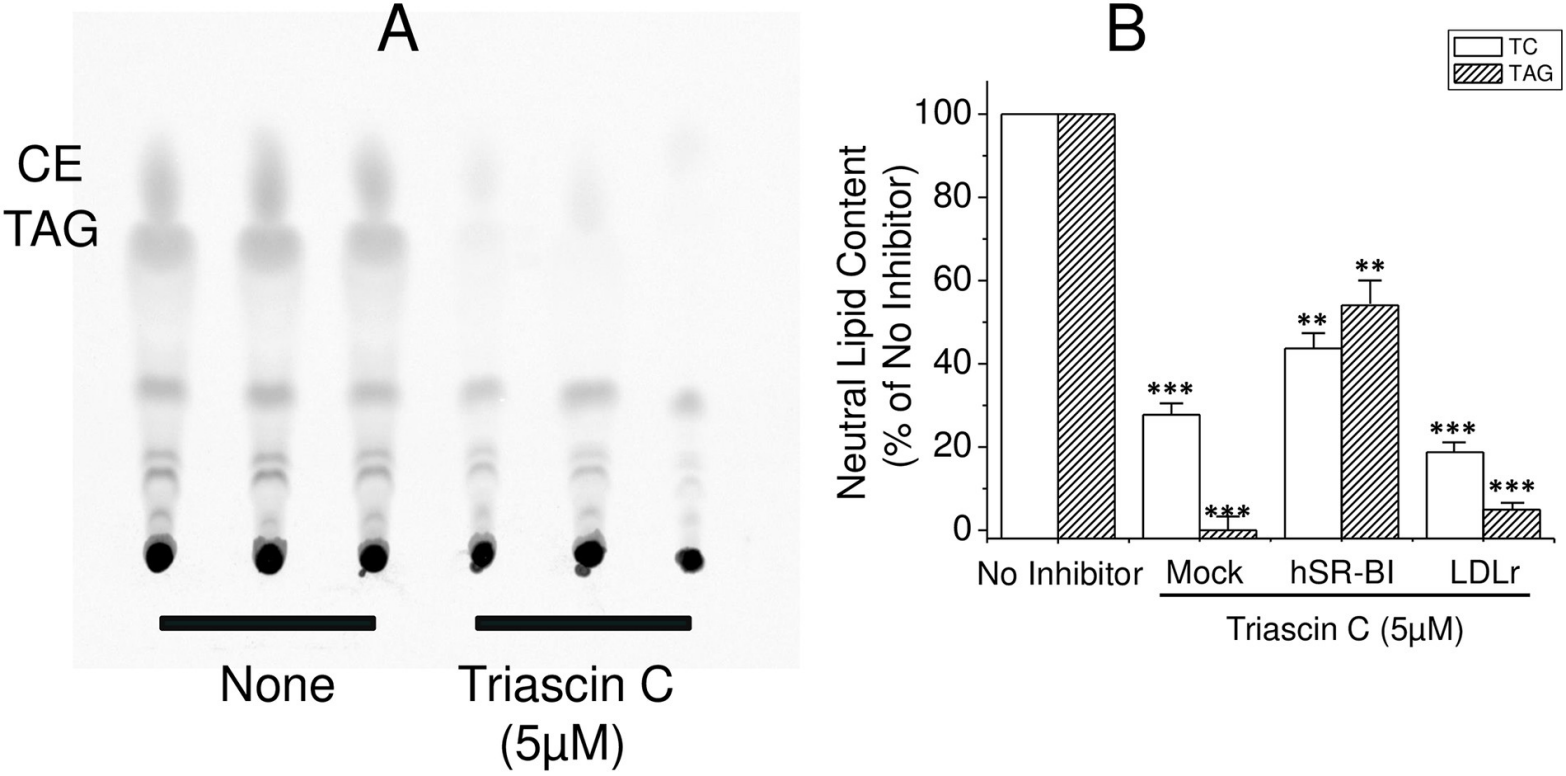
Effect of ACSL inhibitor, Triacsin C, on LD formation in SR-BI-HeLa. SR-BI-HeLa were incubated with 5 μCi/ml of 14C-oleic acid for 6 hours in the presence or absence of 5 μM Triacsin C. After 6 hours of incubation, lipids were extracted and subjected to TLC (Panel A). Alternatively, various HeLa cells were also incubated with or without 200 μg/ml LDL for 6 hours in the presence or absence of 5 μM Triacsin C. Isolated LD TC and TAG were measured by clinical chemistry kits (Panel B). The TC and TAG content was calculated and presented as the percentage of the mean of TC and TAG content in loaded cells in the absence of the inhibitor.

A key enzyme of *de novo* TAG and CE synthesis, ACSL-3, is of most importance for *de novo* LD formation [23], and it was also found to be associated with LDs from SR-BI HeLa [42]. To study the impact of the *de novo* TAG and CE synthesis on LD formation and LDL TAG/CE transport, HeLa cells were loaded with LDL for 6 hours in the presence or absence of 5 μM Triacsin C and ^14^C-oleic acid. Then, LDs were isolated and analyzed for FC, CE and TAG utilizing autography of thin layer chromatography plates as well as utilizing clinical chemistry enzymatical methods when in the absence of ^14^C-oleic acid. As seen in Fig 9A, Triacsin C essentially blocked *de novo* CE and TAG synthesis from ^14^C-oleic acid in SR-BI HeLa cells. It also reduced the number of cellular LDs by 40% at a concentration of 5 μM during a 6-hour loading (S6 Fig). At longer exposures or higher concentrations, Triacsin C demonstrated obvious cytotoxicity leading to cell rounding and detachment. When LD NL content was analyzed utilizing clinical chemistry enzymatic methods, TC and TAG were reduced by 80% and 95%, respectively, in isolated LDs from LDLr-HeLa (Fig 9B). In hSR-BI-HeLa, both were reduced in a lesser extent by about 50%. Furthermore, the inhibition of ACAT (Sandoz 26058) and DGAT (A922500) did not affect LD formation (S6 Fig). In Mock-HeLa, LD TAG and TC were reduced by 98% and 70%, respectively, similar to LDLr-HeLa, (Figs 2 and 9B). This suggests that LD formation by SR-BI includes both direct sorting and transport of LDL neutral lipids to preexisting LDs, processes which do not require *de novo* CE or TAG synthesis. However, simultaneous *de novo* lipid synthesis also occurs further facilitating LD formation.

#### Direct transport of CE and TAG by DESI-MS

**Fig 10:**
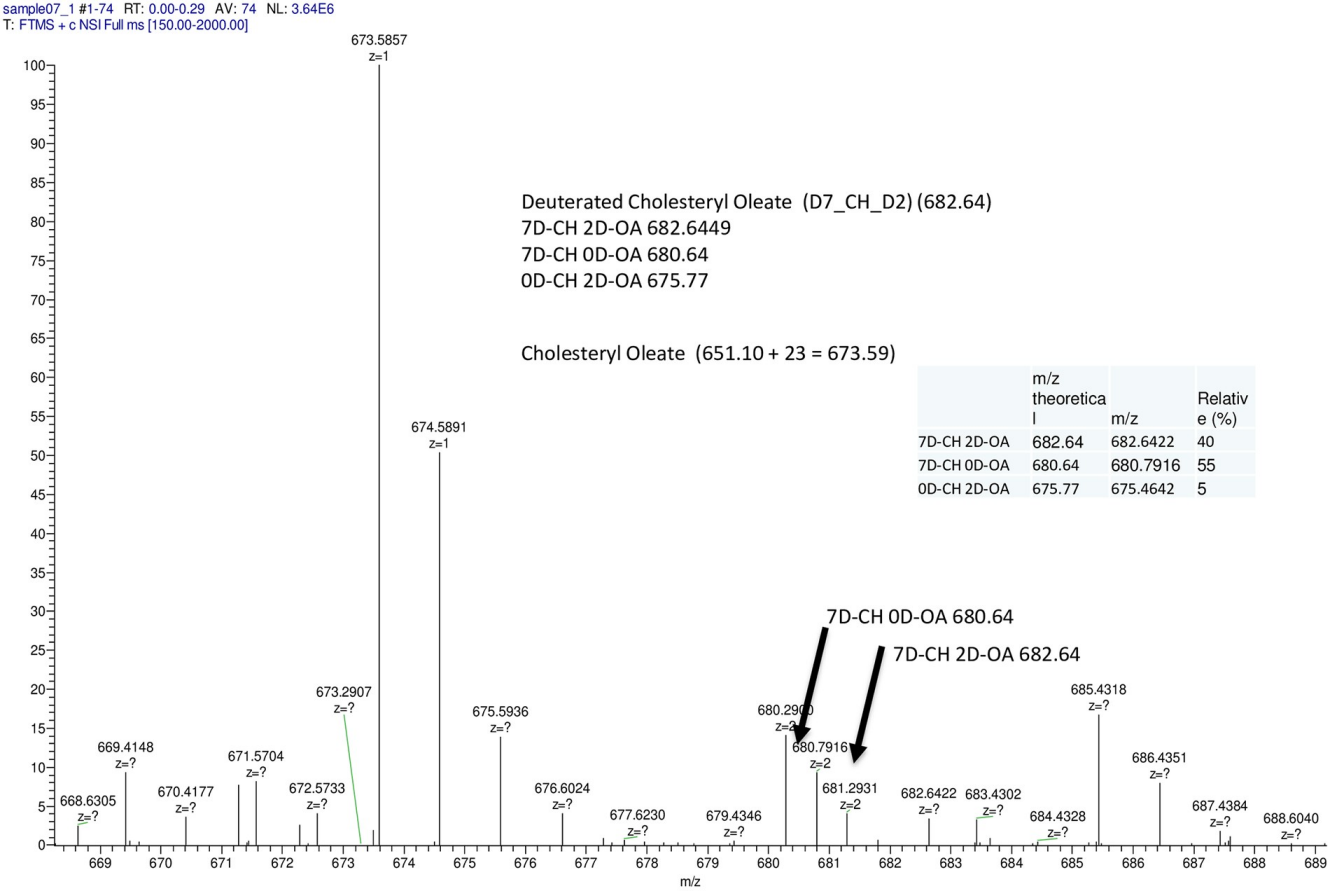
Direct transport of CE as evaluated by ESI-QTOF-MS in SR-BI expressing HeLa cells. The cells were loaded with 200 μg/ml of D9-CE (TO) LDL for 6 hours collected and used for LD isolation. LD lipids were subjected to TLC using banding of the principal neutral lipids isolated and analyzed using ESI-QTOF-MS as described in Materials and Methods. The Insert table describes the three deuterated-CE forms, their spectra and percentiles.

For qualitatively assessing direct versus indirect CE and TAG transport (through *de novo* synthesis CE and TAG versus uploading to LDs), we synthesized D9-cholesteryl oleate, D7-Cholesteryl D2 oleic ester (S7 Fig) and D-5/C-13- triolein (S8 Fig). Using these two derivatives, D9-CE and D-5/13C-TAG labeled LDL were used to upload LDs in SR-BI and LDLr-HeLa cells followed by LD isolation. As seen in Fig 10, up to 40% of D9-CE were directly transported in an unmodified D7-Cholesteryl D2 ester form (MW = 682.64) with about 55% as *de novo* made D7-cholesterol oleic ester product. With respect to deuterated TAG, we could not distinguish unmodified D5/13C-TAG from the D5-TAG product of *de novo* synthesis and therefore could not directly determine an amount of TAG directly transported to LDs (S9 Fig). Longer incubations did affect the distribution, increasing the amount of D7-Cholesteryl oleic ester, further indicating LD hydrolysis affecting NL distribution.

**Fig 11:**
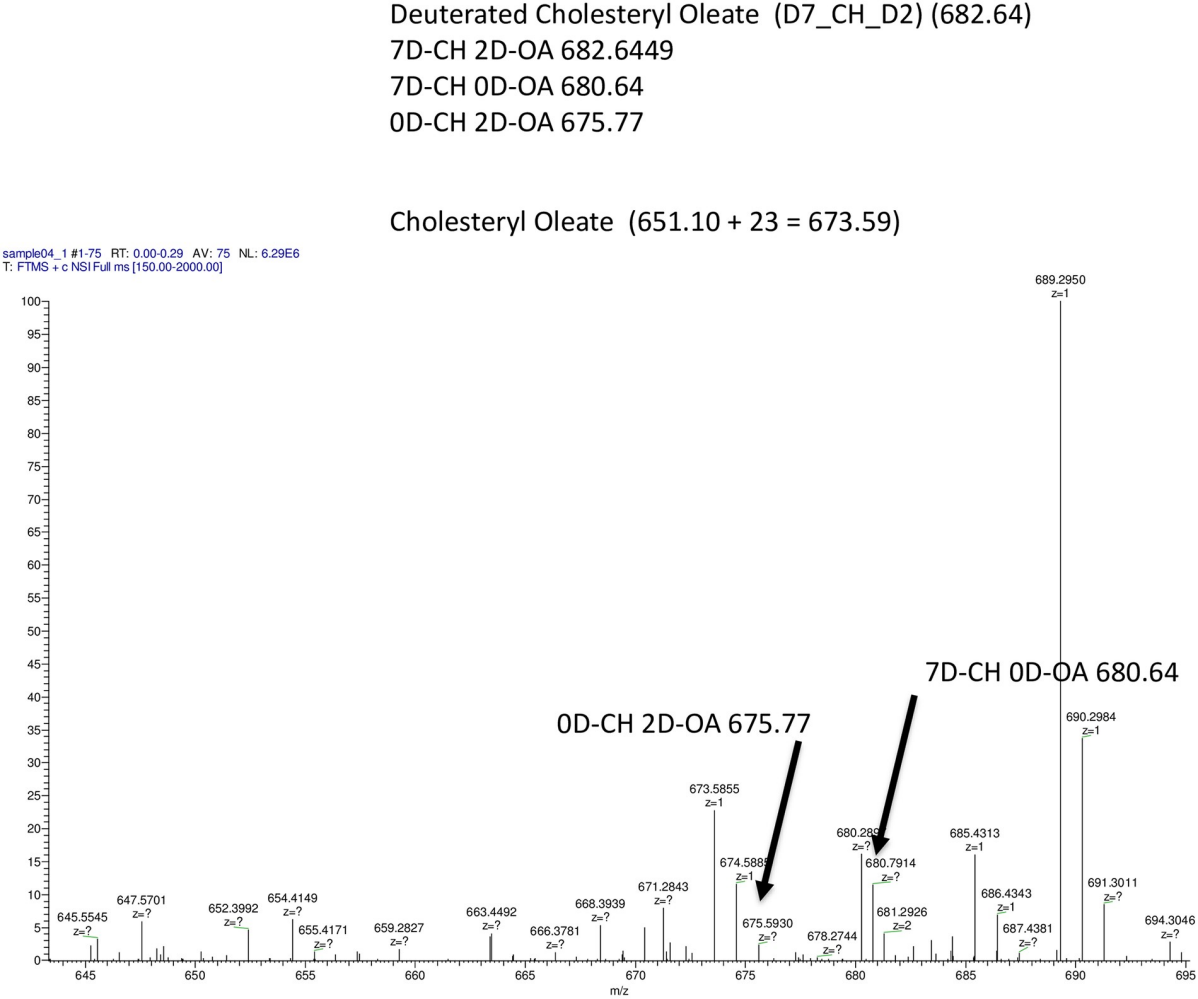
Direct transport of CE as evaluated by ESI-QTOF-MS in LDLr expressing HeLa cells. The cells were loaded with 200 μg/ml of D9-CE (TO) LDL for 6 hours and then collected and used for LD isolation. LD lipids were subjected to TLC using banding of the principal neutral lipids isolated and analyzed using ESI-QTOF-MS as described in Materials and methods.

Uploading D7-Cholesteryl D2 oleic ester LDL in LDLr-HeLa was associated with complete CE hydrolysis with no D7-Cholesteryl D2 ester form detected (Fig 11). Simultaneously, both D7-CE and D2-CE were clearly detectable.

#### Physiological role of SR-BI mediated LD formation *in vivo*

**Fig 12:**
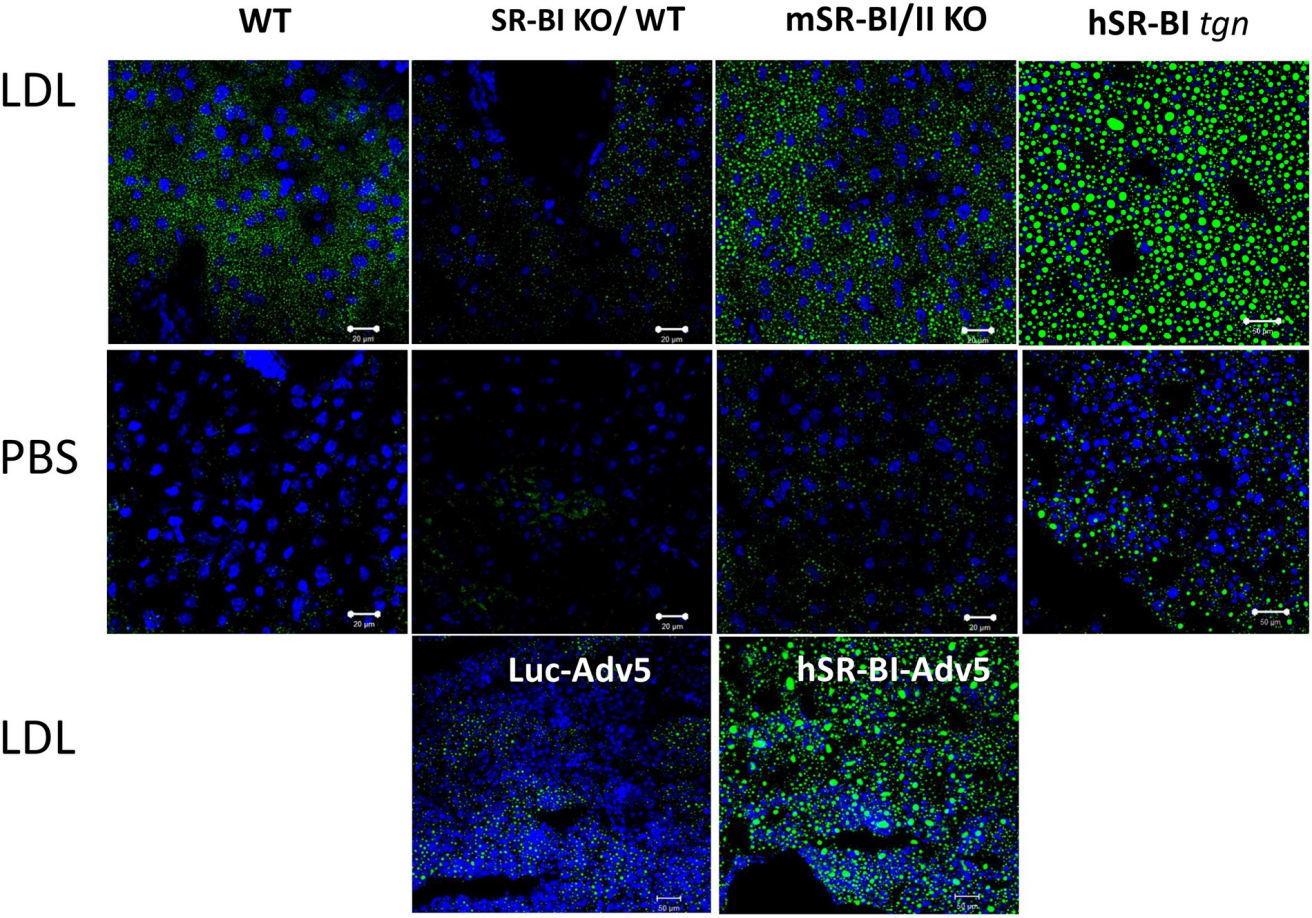
LDL-lipid accumulation in the livers of various mice. Mice were fasted overnight and injected IV with either PBS (middle panel) or 2 mg of human LDL (upper and lower panels) and sacrificed 2 hours later. The upper two rows of panels show LD staining in liver cryosections from normal, SR-BI/II KO, hSR-BI transgenic SR-BI/II KO and hSR-BI transgenic normal mice after (+) or without (-) an LDL injection. The lower panels show LD staining in liver cryosections of Luc-AdV5 and SR-BI-AdV5 treated SR-BI KO mice following LDL injection. The animal codes are written on the panels. The bar corresponds to 20 μm.

The *in vivo* role of SR-BI was assessed in livers from wild type (WT), SR-BI KO and SR-BI transgenic mice receiving an intravenous bolus of 2 mg of human LDL or saline solution. After overnight fasting, PBS injected mSR-BI/II KO, hSR-BI transgenic over WT as well as SR-BI/II KO background and WT mice demonstrated relatively low LD staining in all liver sections (Fig 12 middle panels). Two hours after an LDL bolus, a significant accumulation of LDs was seen in livers from normal mice but little LD staining was found in SR-BI KO mice. Both types of hSR-BI transgenic mice demonstrated an even larger amount of LD formation than normal mice (Fig 12 upper panels). Human SR-BI *tgn* over WT transgenic mice demonstrated larger LDs when compared to hSR-BI *tgn* over SR-BI KO mice, which we attribute to a higher total SR-BI content leading to higher NL accumulation rather than a direct effect of SR-BI on LD structure. As in our *in vitro* studies (S4 and S5 Figs) where LDs contained PLIN2 and 3, liver LDs exhibited PLIN3 and PLIN2 but less PLIN1 expression as assessed by immunostaining using corresponding antibodies (S10–S12 Figs). Further confirming the relevance of SR-BI-mediated LD formation, SR-BI/II KO mice were transfected with hSR-BI and Luciferase expressing AdV5. LD accumulation was restored by hSR-BI-AdV5, but not by control Luc-AdV5 (Fig 12 lower panels).

## Discussion

LDs are intracellular cytosolic organelles that accumulate neutral lipids (NL) including CE and TAG [4, 43]. NL are derived from both endogenous (*de novo* lipid synthesis) and exogenous (lipoprotein receptor mediated lipoprotein uptake) sources [41]. This paper primarily studied the role of two lipoprotein receptors (LDLr and SR-BI), utilizing exogenous lipoprotein-derived NLs for LD formation *in vitro* and *in vivo*. SR-BI, a lipoprotein receptor that binds various lipoproteins including chylomicrons, VLDL, LDL and HDL, was the major target of the study [13, 44]. It has been reported that after various lipoprotein binding to SR-BI, apolipoproteins and NL (TAG and CE) can be internalized and sorted to various compartments [45–47], including LDs, late endosomes and to a lesser extent lysosomes [48]. Despite a well investigated mechanism for the selective HDL-CE uptake [44, 49], and the importance of SR-BI in formation of adrenal CE storage [18, 23], relatively little is known about SR-BI-mediated LDL TAG and CE transport to LDs and its role in LD formation in the liver. In this paper, we compared mechanisms of LDL CE and TAG transport mediated by SR-BI with classical LDL endocytosis mediated by the LDL receptor, and investigated their relative roles in LD formation.

As in previous reports [49] we have found that SR-BI functions as an LDL receptor, mediating similar dose dependent ^125^I-LDL and Alexa 488-LDL uptake in HeLa cells. When comparing stably transfected HeLa cell lines, both hSR-BI- and hLDLr-HeLa cells demonstrated increased Alexa 488- and ^125^I-LDL uptake when compared to mock-HeLa (Fig 1) with the uptake modestly higher (about 20–30%) in SR-BI versus LDLr-HeLa. In comparison to the significantly increased LDL degradation associated with the hLDLr, hSR-BI overexpression only slightly increased LDL degradation in hSR-BI-HeLa which is comparable to previously reported data [50]. In contrast, LDL loading increased LD formation in SR-BI-HeLa to a much greater extent than either LDLr-HeLa or mock-HeLa (Fig 2A). The increase in LDs was assessed by *in situ cell* staining with neutral BODIPY, TLC (S3 Fig) and clinical chemistry analysis of NL in purified LDs. Utilizing these methods, we have found that LDs from SR-BI-HeLa contained 2.5–5 times more TC and TAG than mock-HeLa. We have hypothesized that LDL uptake could facilitate not only the previously recognized LDL CE selective uptake [11] but also LDL’s CE and TAG transport and accumulation in and expansion of LDs. This is consistent with previously reported data that SR-BI mediates selective uptake of both HDL ^14^C-triolein and ^14^C-CE [22]. We considered two mechanisms of LD formation driven by hSR-BI including a direct NL transport to LDs as well as a two-step process associated with NL hydrolysis followed by *de novo* NL synthesis and an accumulation of newly synthesized NL in LDs. However, since an analogue of CE, BP-CE from both HDL [19] and LDL (Fig 6) accumulate in LDs in an SR-BI-dependent manner, it seemed feasible that both CE and TAG could also be directly transported to LDs facilitating their formation and expansion. The preparations of LDs isolated from hSR-BI-HeLa were characterized by the presence of TIP47 (PLIN3), adipophilin (PLIN2) and long chain fatty acid synthetase, ACSL-3 (S4 and S5 Figs), but not PLIN1, which clearly distinguish hSR-BI-HeLa-derived from adipocyte LDs. For analyzing early and late steps of LDL apolipoprotein and lipid uptake, such as receptor LDL binding, lipid sorting, internalization and CE/TAG transport to LDs, we used Alexa 488 (or 568), BODIPY-CE, or BODIPY-TAG labeled LDL or their combinations of dual labeled LDL (see S1 and S2 Figs). The LDL labeled with fluorescent analogues of TAG and CE were prepared similarly to the initial technique introduced by Azar et al. for HDL labeling and further modified by us for multi-labeling LDL [51]. Using variously labeled LDL, we found that SR-BI mediated selective sorting of both LDL CE and TAG from apolipoprotein containing LDL particles and facilitated their internalization to LDs without substantial accumulation in lysosomes. This observation is in agreement with the general understanding of SR-BI function, where selective HDL CE uptake is not associated with HDL degradation [52, 53]. In LDLr-HeLa, LDLr mediated holoparticle uptake and endocytosis to the lysosomal compartment which was associated with 2-3-times smaller TAG and CE accumulation in LDs (3-6-hour incubation) than in hSR-BI-HeLa. This indicates that LD formation occurs through both the LDLr- and SR-BI- dependent mechanisms. The LDLr-dependent pathway appears important for longer term LD formation through acid lysosomal hydrolysis of LDL neutral lipids followed by *de novo* TAG and CE synthesis in the ER facilitating newly LD formation. In contrast, SR-BI could facilitate rapid and direct BP-TAG and BP-CE transport into LDs without lipid modifications. This is confirmed by the very modest LDL endocytosis to lysosomes in SR-BI-HeLa, indicating that both CE and TAG are primarily sorted to LDs without the intermediate steps of acid lysosomal lipid hydrolysis or TAG/CE de novo synthesis.

Involvement of a cytosolic neutral esterase mediating CE cytosolic hydrolysis followed by ACAT catalyzed cholesterol esterification and *de novo* formation of LDs was also suggested as an important mechanism for CE transport and LD formation in the adrenal gland [11, 23]. In mouse adrenal cells practically all LD formation was HSL (a ubiquitous neutral CE and TAG hydrolase)-dependent [11, 23] but not in the liver which demonstrates a low HSL level. However, the role of human HSL as the major contributor to LD formation remains controversial because of the low HSL expression in adrenal gland [54]. However, an inhibition of ACSL-3 by Triacsin C reduced LDs significantly and decreased LD CE and TAG by 50% in SR-BI-HeLa and by more than 90% in LDLr-HeLa. This demonstrates that both *de novo* synthesis and direct CE and TAG transport to LDs take place during SR-BI-dependent LD formation. Considering relatively low TAG content of LDL (8% compared to 42% CE), we also suggest that a major part of accumulated TAG resulted from SR-BI dependent LDL stimulated endogenous TAG synthesis which comprises most of accumulated LD TAG. This was not a subject of this study and will be investigated later.

To confirm direct CE and TAG transport to LDs, we also used deuterated D9-CE and D5/13C-TAG labeled LDL to upload LDs (S7 and S8 Figs). Lipids extracted from isolated LDs were subjected to TLC, bands cut off and extracted lipid subjected to mass spectral analysis. We found that up to 40% of D9-CE (Fig 10) was delivered to LDs without hydrolysis which is in agreement with Triacsin C inhibition data indicating that up to 50% of CE accumulation was inhabitable by Triacsin C (Fig 9). Since deuterated 9D-CE is found in only a tracer amount (less than 1/1000 of endogenous CE), the presence of 9D-CE cannot result from hydrolysis and de novo synthesis from 2D-oleic acid and 7D-cholesterol. However, while Triacsin C also reduced TAG accumulation by 50% in SR-BI-HeLa LDs, we could not estimate amounts of D5/13C-TAG in LDs because there was only a 1 Dalton-difference between D5-TAG (*de novo* synthesis) and D5/13C-TAG (unchanged) forms which was the limit of MS detection. Although the measurement of direct transport of TAGs to LDs without hydrolysis was not determinable like CE, from the similarity of the Triacsin C inhibition data and very modest LDL endocytosis to lysosomes in SR-BI-HeLa we believe that like CE, up to 40% of TAG could be directly transported from LDL to LDs.

We propose a model of lipoprotein sorting and selective neutral lipid accumulation in LDs (Fig 9). In classical LDLr endocytosis, LDL is internalized to lysosomes and degraded while the receptor is recycled to the cell surface. Free cholesterol, fatty acids and glycerol are used for TAG and CE synthesis and LD formation in the ER. In SR-BI expressing cells, after the initial LDL binding to SR-BI, some of the CE and TAG are sorted from bound LDL at cell plasma membrane microdomains and internalized through a yet to be determined mechanism to LDs. Simultaneously, some newly synthesized CE and TAG from intracellular, SR-BI derived, hydrolyzed CE and TAG also accumulate in LDs further expanding their size. TAG and CE are sorted at the plasma membrane and form small (50–100 nm) endocytic SR-BI/apoB free vesicles that transport TAG and CE to LDs. This NL endocytosis was found to be clathrin, dynamin and caveolin independent suggesting a novel endocytic pathway which is now under investigation in our laboratory. However, we believe that this mechanism might be similar to the SR-BI HDL BP-CE uptake mechanism which we reported previously (ATVB, 2013, Vol: 33, No. suppl. 1, abstract 66). In that report we found that tetraspanin enriched microdomains (TED) function as CE sorting sites with CD81 helping to form nano-vesicles that deliver CE to LDs.

To establish the physiological relevance of SR-BI mediated LD formation *in vivo*, we analyzed LD formation in mice and found that SR-BI is important in the rapid expansion of LDs in the liver of LDL loaded mice. Previously, studies investigated the role of SR-BI in plasma lipoprotein metabolism. It was demonstrated that VLDL and chylomicron metabolism were critically dependent on liver SR-BI, because SR-BI KO mice exhibited greatly reduced VLDL and CM liver uptake [44]. With adenoviral SR-BI transfection, VLDL and chylomicron plasma lipids were readily normalized in SR-BI KO mice [55, 56]. Similarly, hSR-BI adenoviral expression has been also reported to reduce neutral lipids in plasma LDL, VLDL and HDL fractions of wild type mice with an unknown effect on plasma apoB protein levels [57]. In contrast to lipoprotein profiles and plasma lipid pattern distribution [58], there is limited data for the role of SR-BI in tissue lipid accumulation and LD formation *in vivo*. Some of those studies have demonstrated that SR-BI overexpression is associated with increased lipid accumulation [55] in the liver. Results from our study revealed that SR-BI is critically important for LD formation in the mouse liver (Fig 12). Indeed, SR-BI/II KO mice demonstrated a dramatically reduced ability to accumulate LDs in liver tissue (Fig 12). Moreover, when SR-BI/II deficiency was restored in SR-BI/II KO mice using adenoviral hSR-BI transduction, LD formation and neutral lipid accumulation was normalized relative to the Luc-adV5 control (Fig 12 lower panel). Human SR-BI transgenic mice accumulated markedly more LDs than normal mice after an LDL intravenous bolus and had a LD pattern similar to that of fatty liver. Immunostaining of mouse liver LDs revealed that like in SR-BI-HeLa, normal and hSR-BI transgenic liver LDs express PLIN3, PLIN2 and a lesser amount of PLIN1 (S10–S12 Figs) which is very similar to human liver [59] and can potentially provide an important humanized mouse model for studying liver lipid disorders. All together, these data indicate that SR-BI plays a central role in the tissue regulation of cellular LD formation and neutral lipid accumulation both in cultured cells and the liver.

In conclusion, this study demonstrates that SR-BI mediates LDL binding, CE and TAG sorting followed by their internalization to LDs. While about 60% of LDL delivered NL to be uploaded to LDs require neutral hydrolysis and *de novo* neutral lipid synthesis, about 40% is directly sorted and transported to LDs. Most importantly, both *in vitro* and *in vivo* data suggest that SR-BI is critical for short term LD formation from LDL in the liver and potentially other organs.

## Supporting information

S1 Fig LDL labeling tracers. (TIF)

S2 Fig LDL labeling strategy. (TIF)

S3 Fig Lipid analyses of LDs isolated from various HeLa cells. (TIF)

S4 Fig Mass spectrometry of LDs isolated from hSR-BI overexpressing cells. (TIF)

S5 Fig Analysis of the purity of LDs isolated from hSR-BI- and LDLr-expressing HeLa cells. (TIF)

S6 Fig Effect of ACAT, ACSL and DGAT inhibitors on LD formation in SR-BI-HeLa cells. (TIF)

S7 Fig Structure, molecular weight and DESI-MS spectra for d9-CE. (TIF)

S8 Fig Structure, molecular weight and DESI-MS spectra for D5/C13-TAG. (TIF)

S9 Fig Analysis of deuterated TAG purified from the LD fraction of HeLa SR-BI treated with D5,C13-TAG labelled LDL. (TIF)

S10 Fig LD PLIN3 (TIP47) protein staining in liver sections of hSR-BI transgenic mice receiving intravenous PBS (upper panels) or LDL bolus (lower panels). (TIF)

S11 Fig LD PLIN2 (adipiphilin) protein staining in liver sections of hSR-BI transgenic mice receiving intravenous PBS or LDL bolus. (TIF)

S12 Fig LD PLIN1 (Perilipin) protein staining in liver sections of hSR-BI transgenic mice receiving intravenous PBS or LDL bolus. (TIF)

S1 Raw images (PDF)
