# Supplementary figures and images for: SR-BI mediates neutral lipid sorting from LDL to lipid droplets and facilitates their formation
Source: PLoS One. 2020 Oct 15;15(10):e0240659. doi: 10.1371/journal.pone.0240659 (PMC7561250; doi:10.1371/journal.pone.0240659)

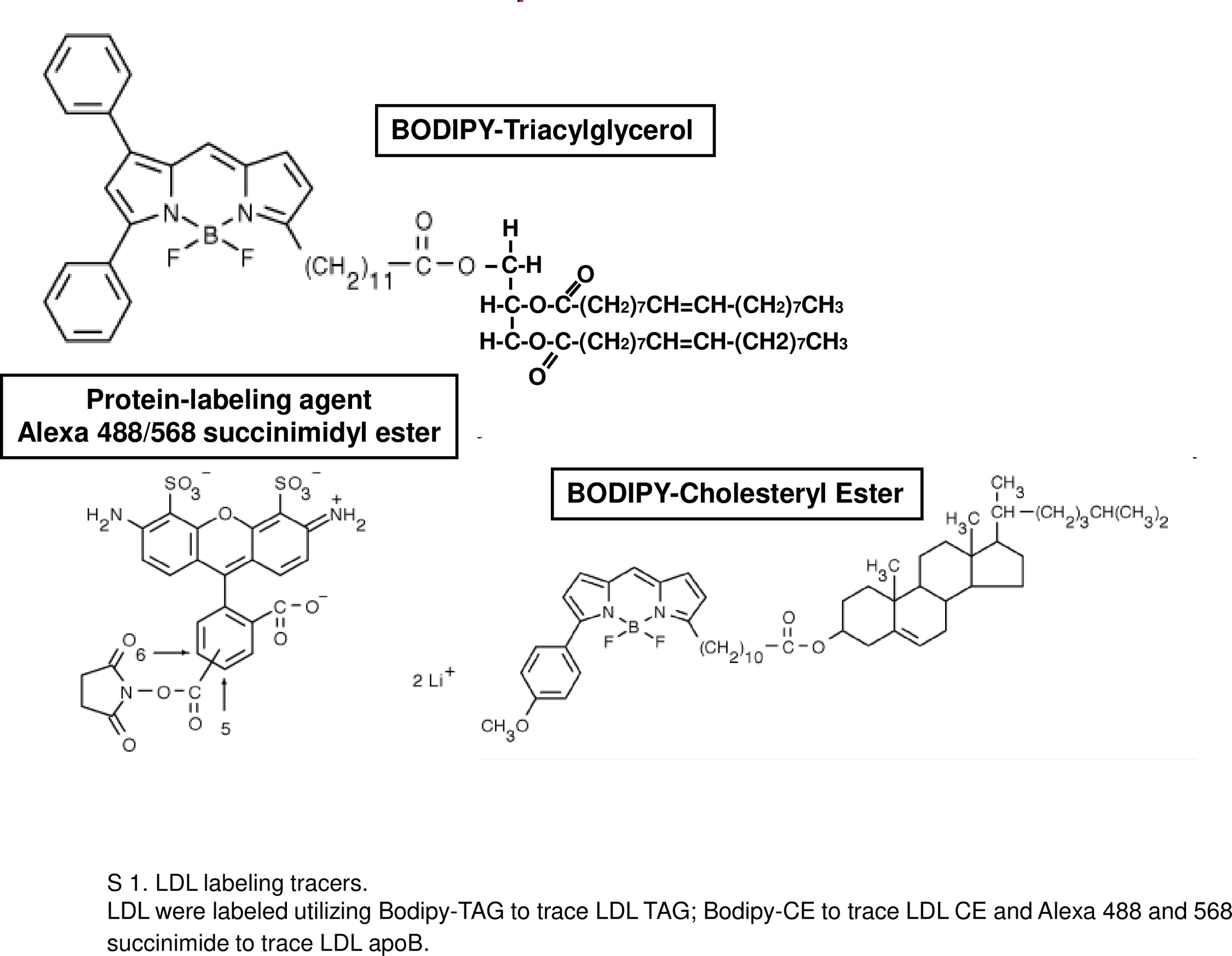

Supplement: S1 Fig — (TIF) [file pone.0240659.s001.tif]

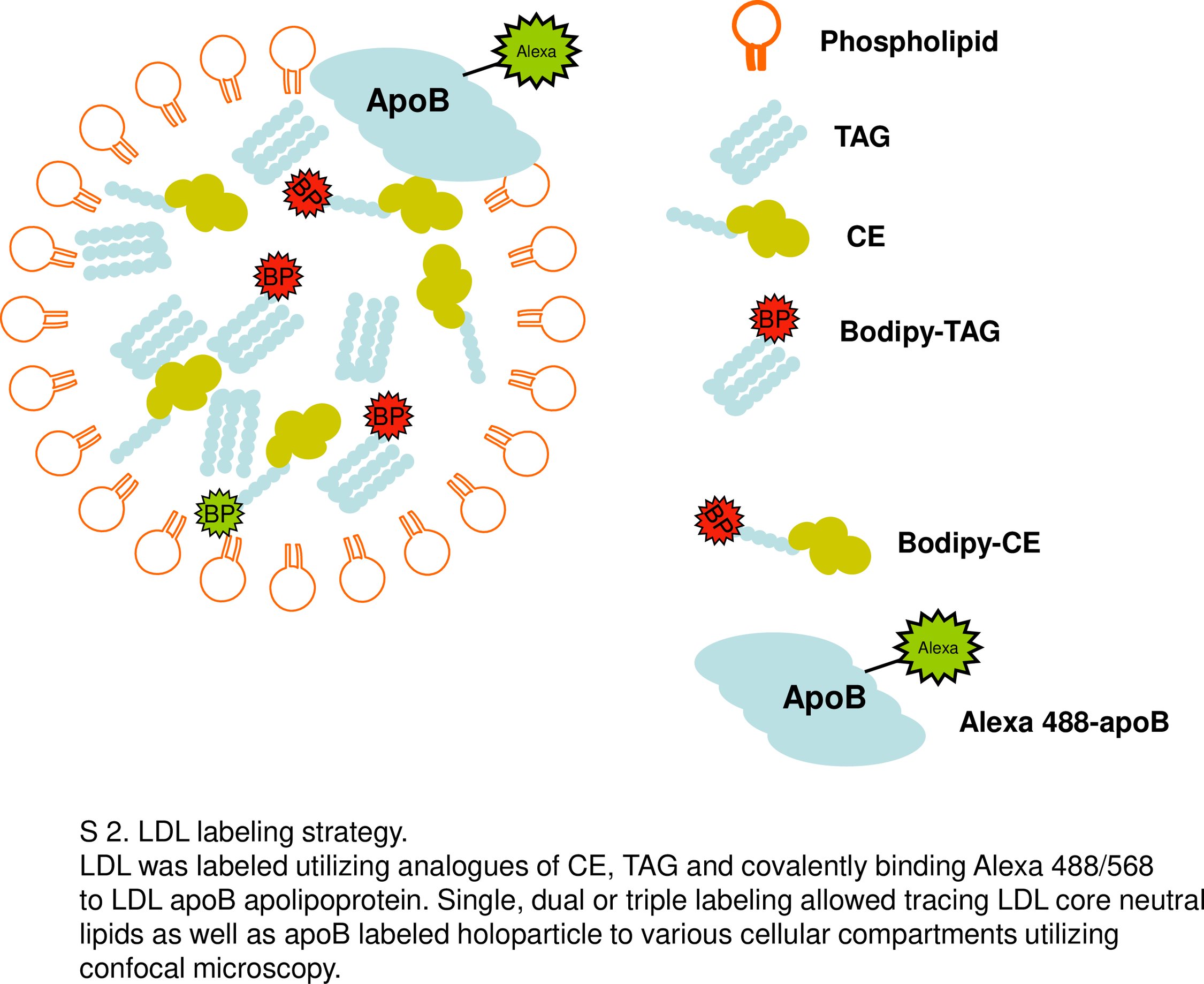

Supplement: S2 Fig — (TIF) [file pone.0240659.s002.tif]

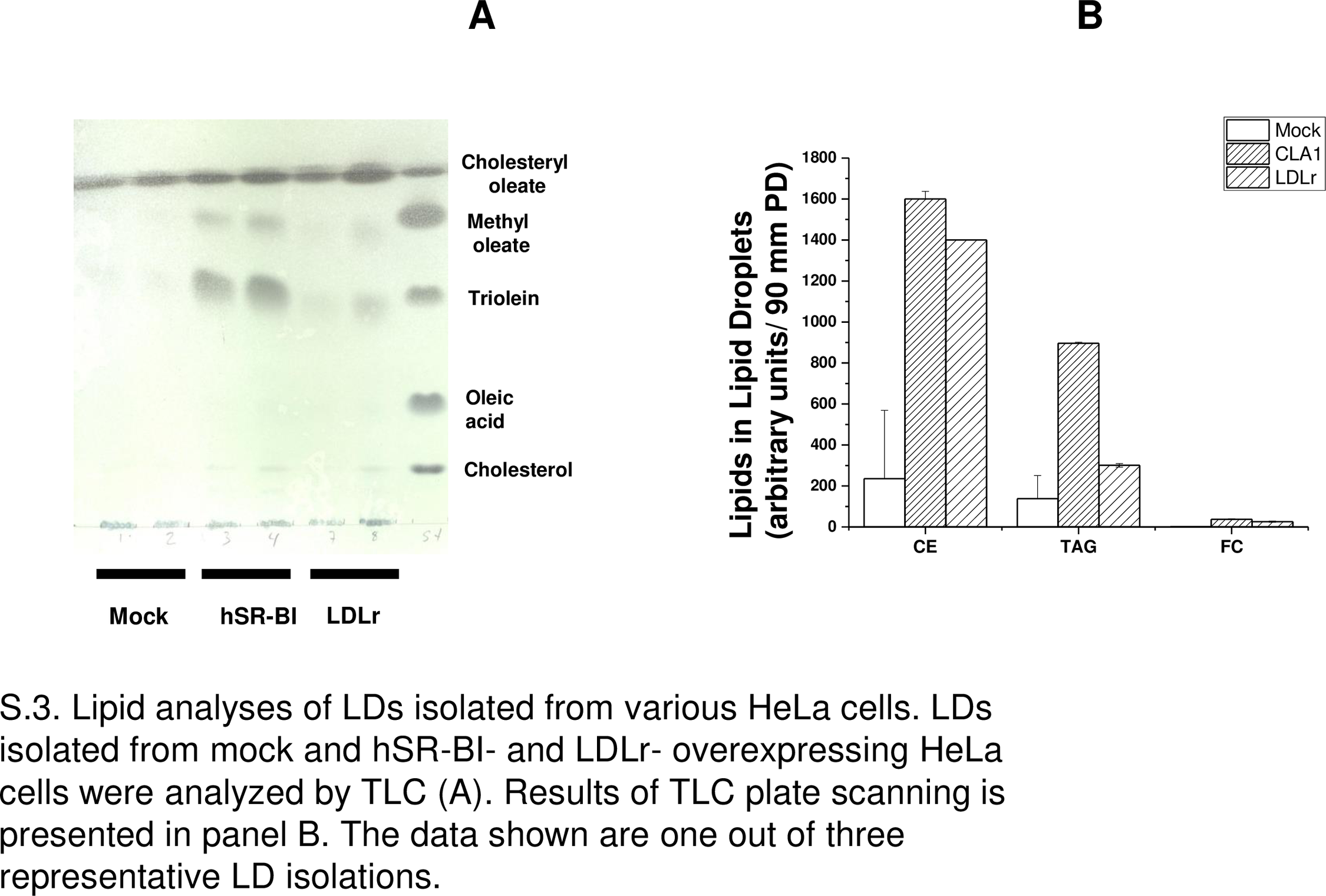

Supplement: S3 Fig — (TIF) [file pone.0240659.s003.tif]

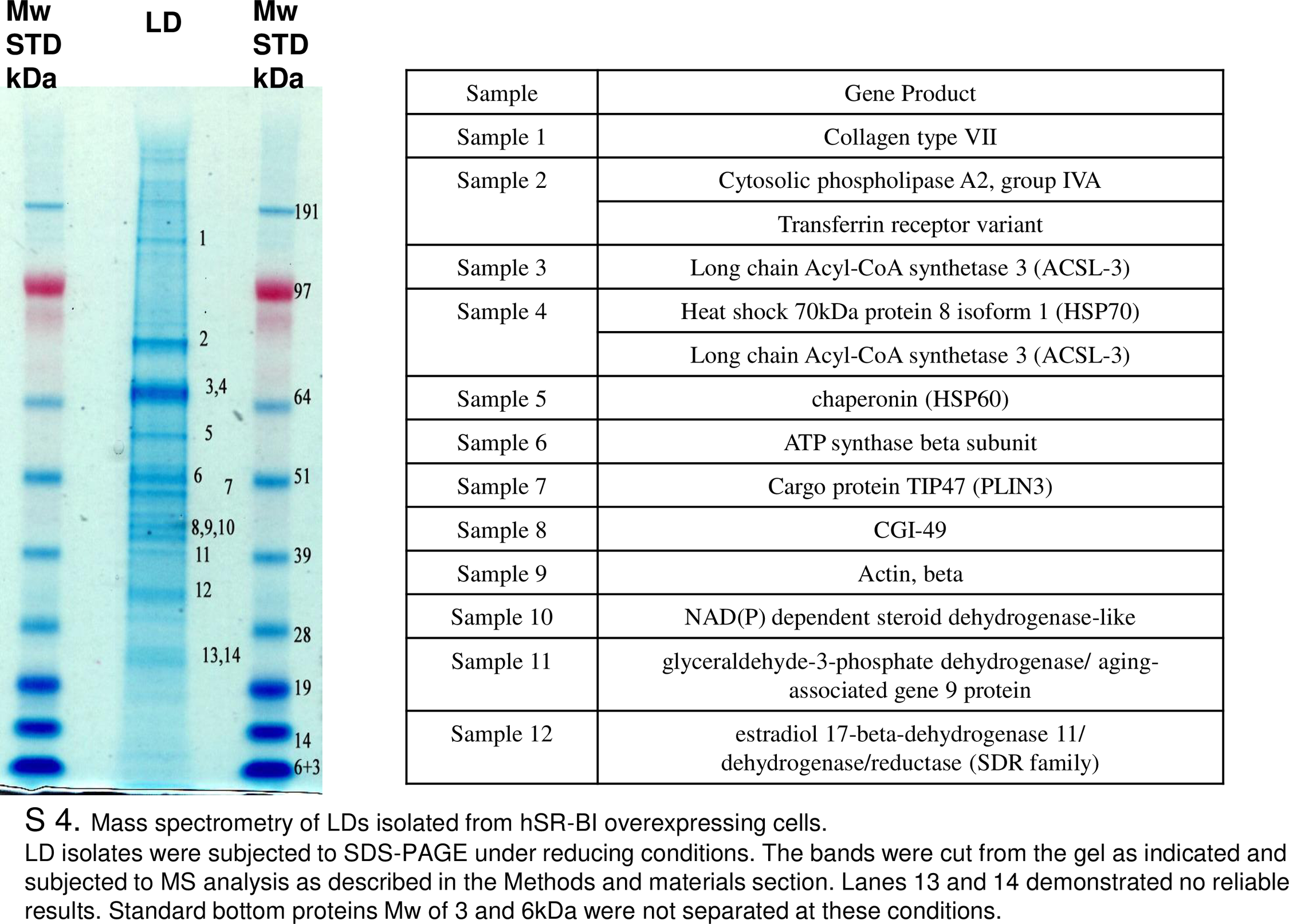

Supplement: S4 Fig — (TIF) [file pone.0240659.s004.tif]

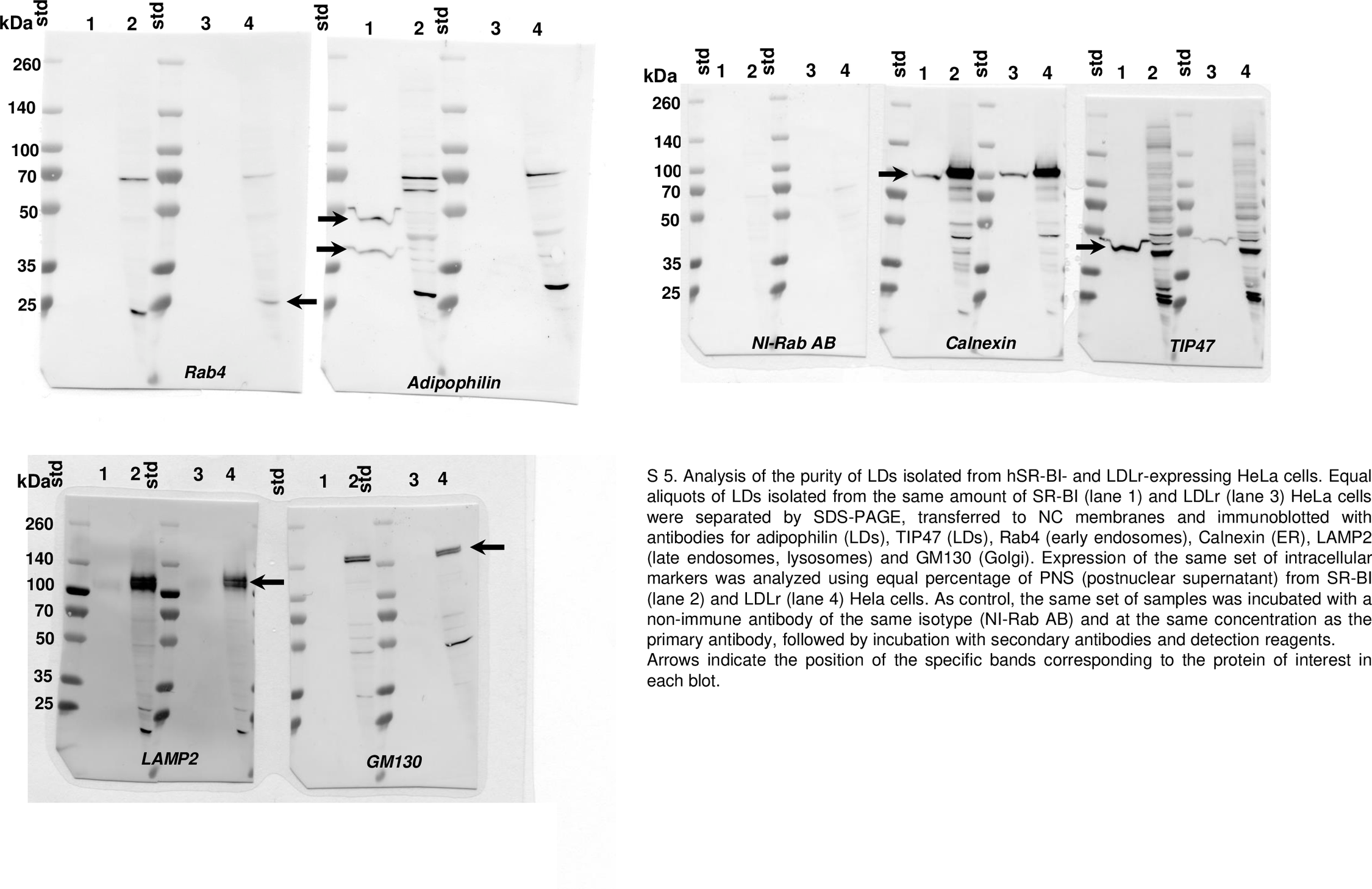

Supplement: S5 Fig — (TIF) [file pone.0240659.s005.tif]

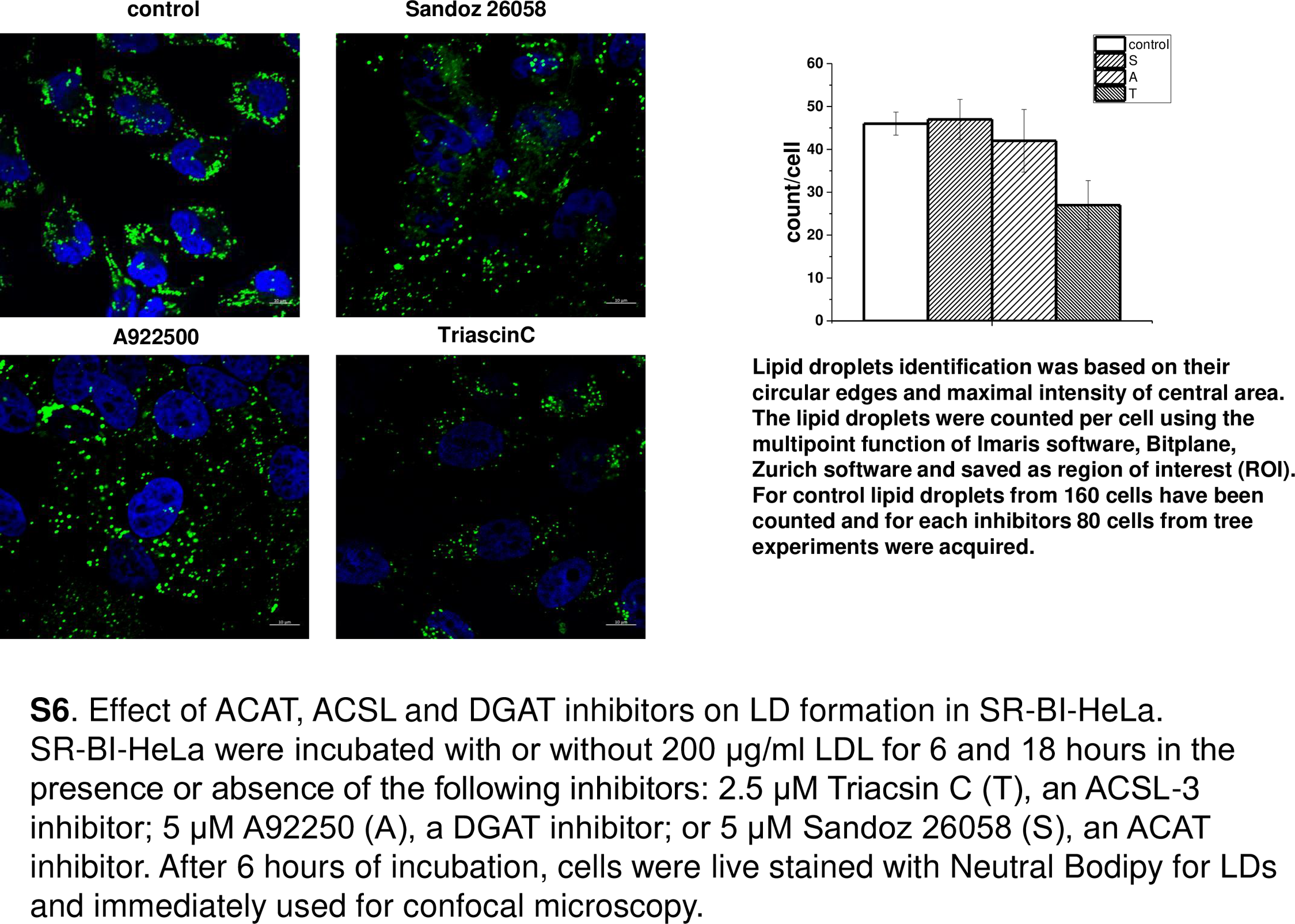

Supplement: S6 Fig — (TIF) [file pone.0240659.s006.tif]

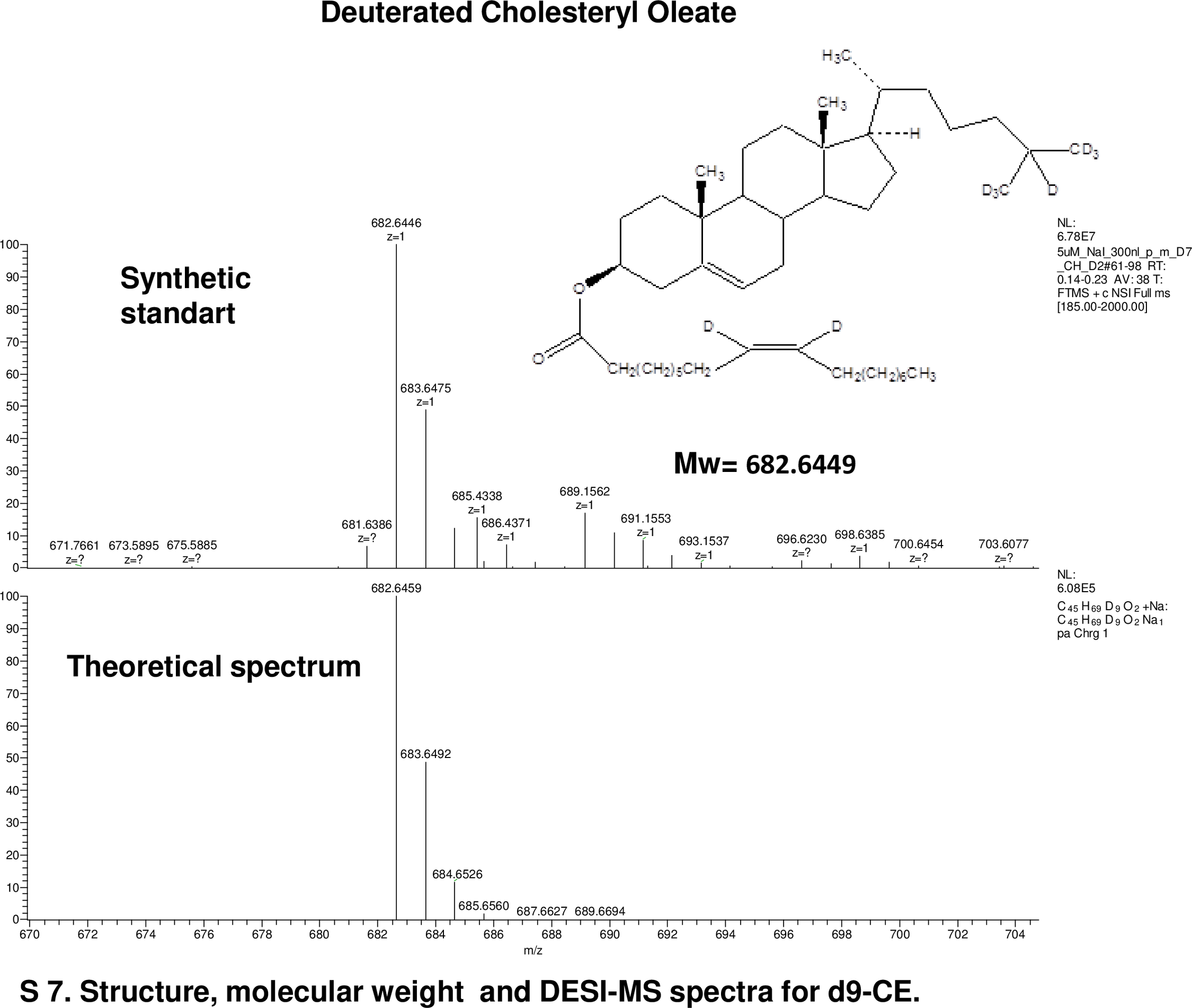

Supplement: S7 Fig — (TIF) [file pone.0240659.s007.tif]

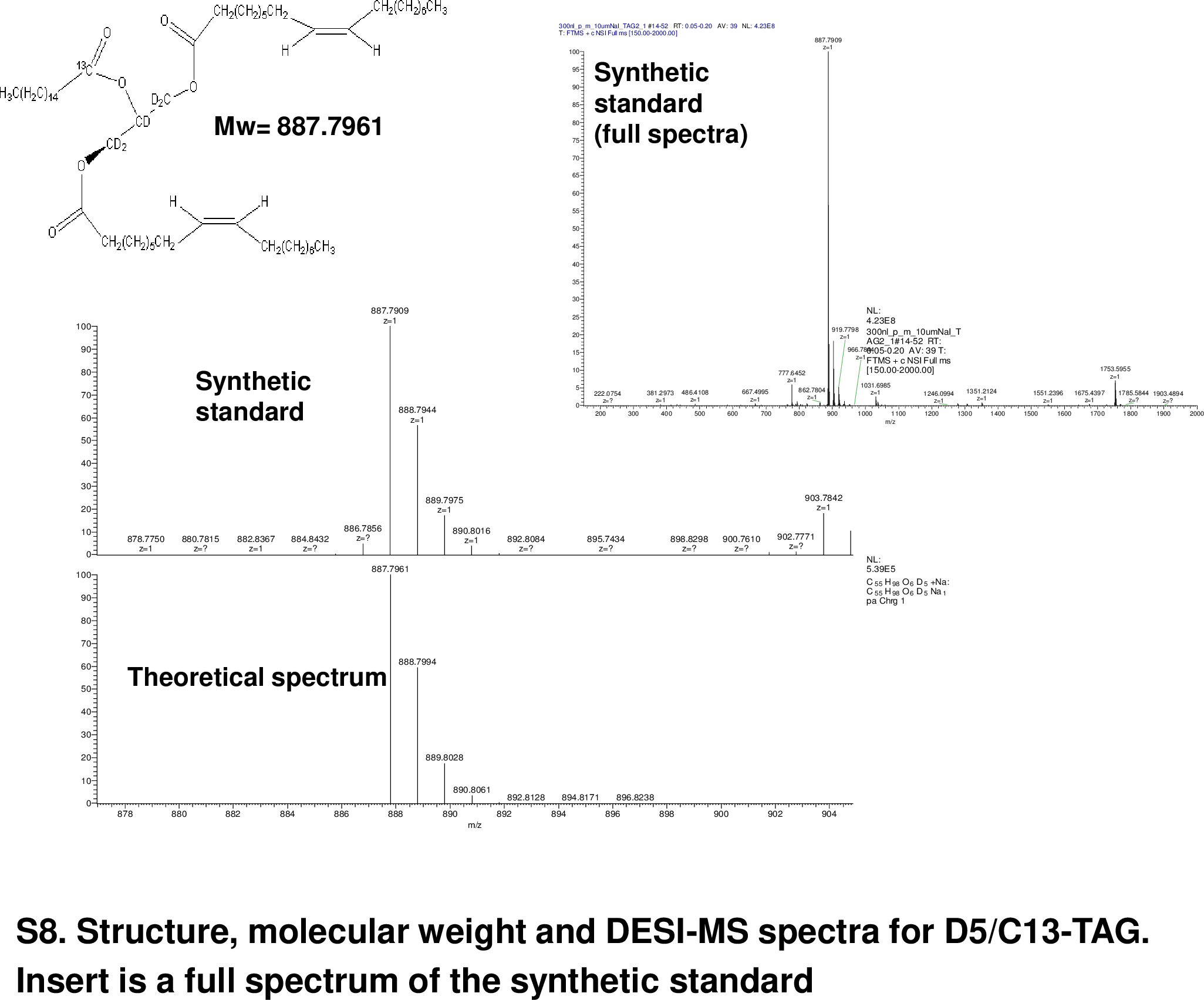

Supplement: S8 Fig — (TIF) [file pone.0240659.s008.tif]

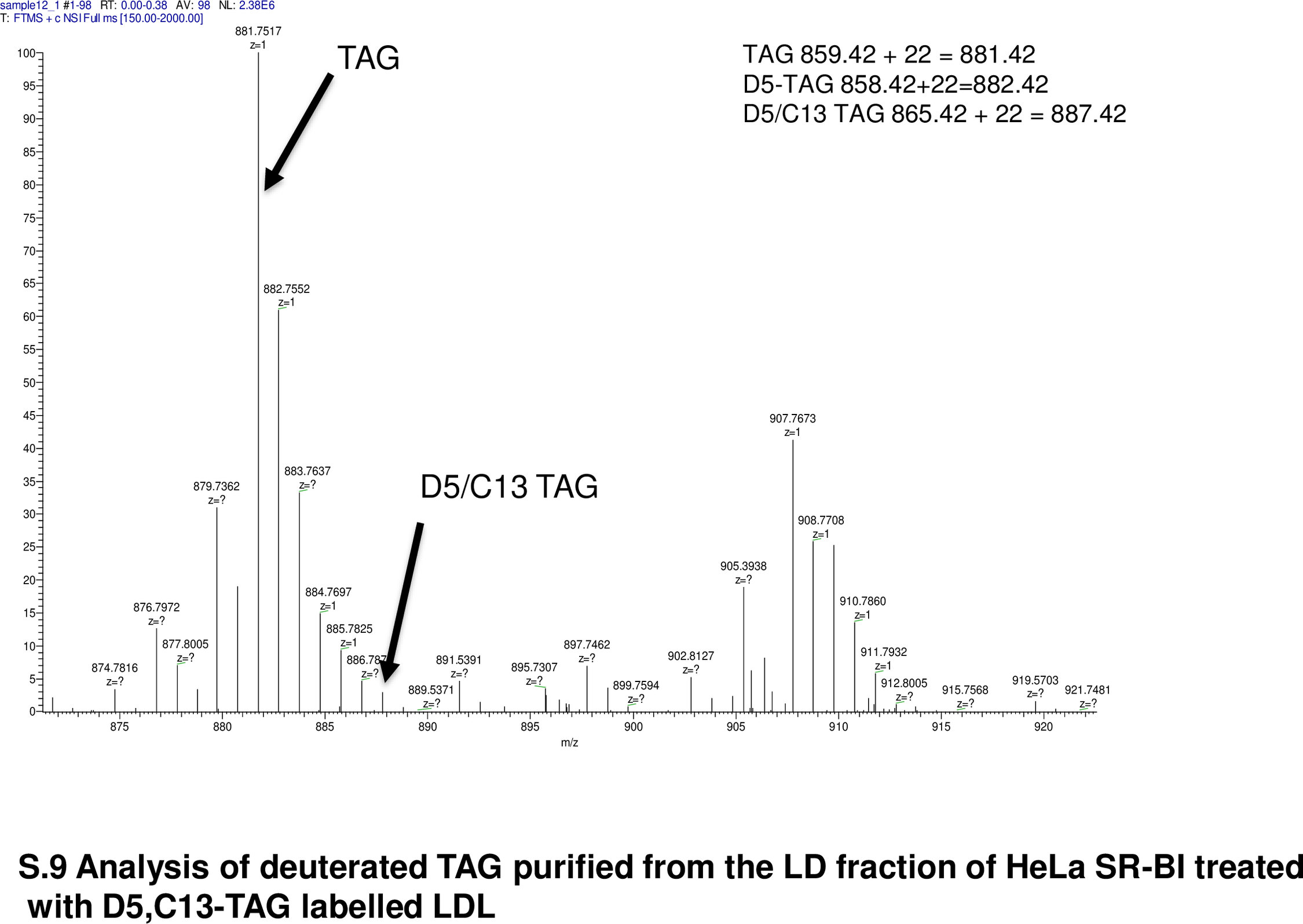

Supplement: S9 Fig — (TIF) [file pone.0240659.s009.tif]

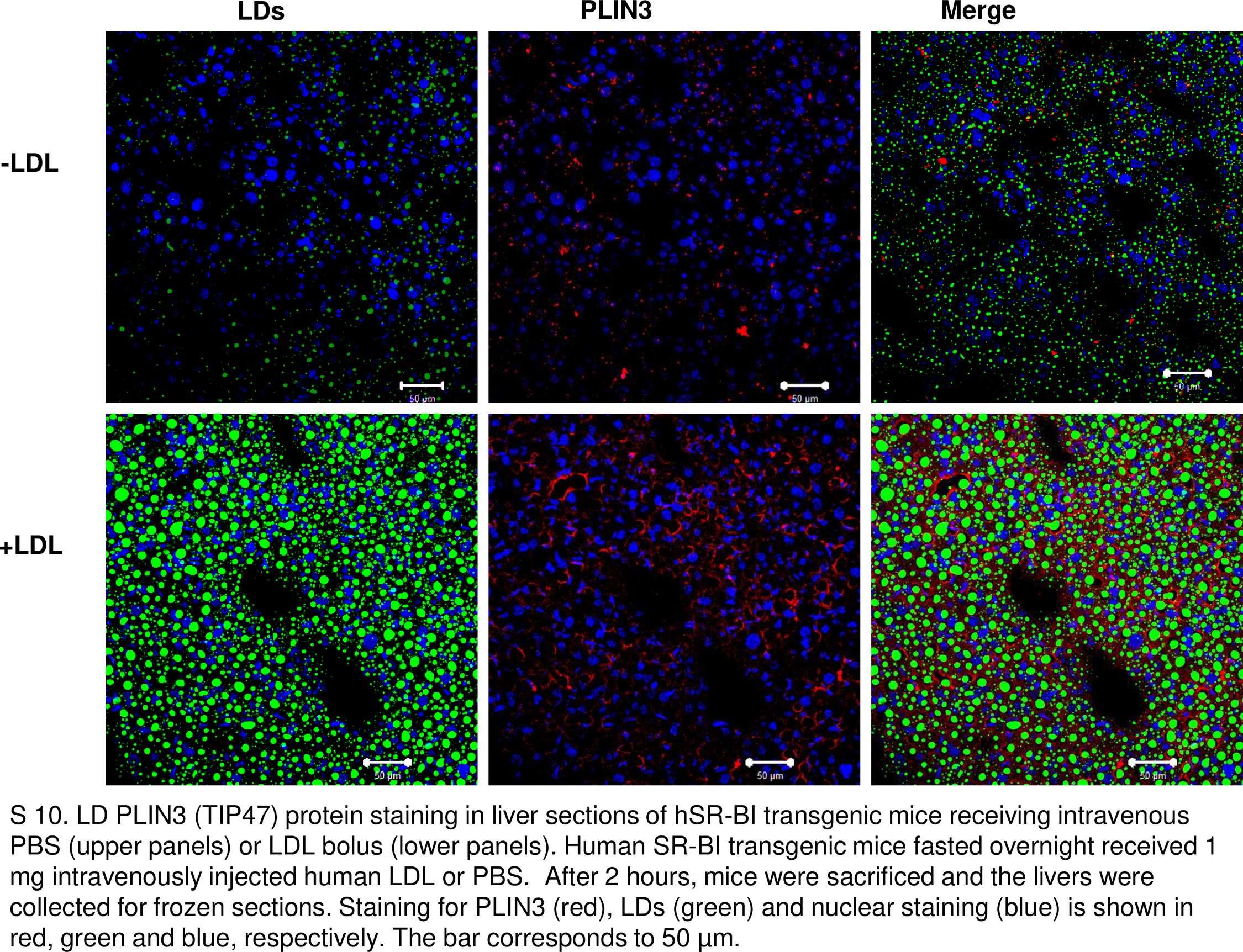

Supplement: S10 Fig — (TIF) [file pone.0240659.s010.tif]

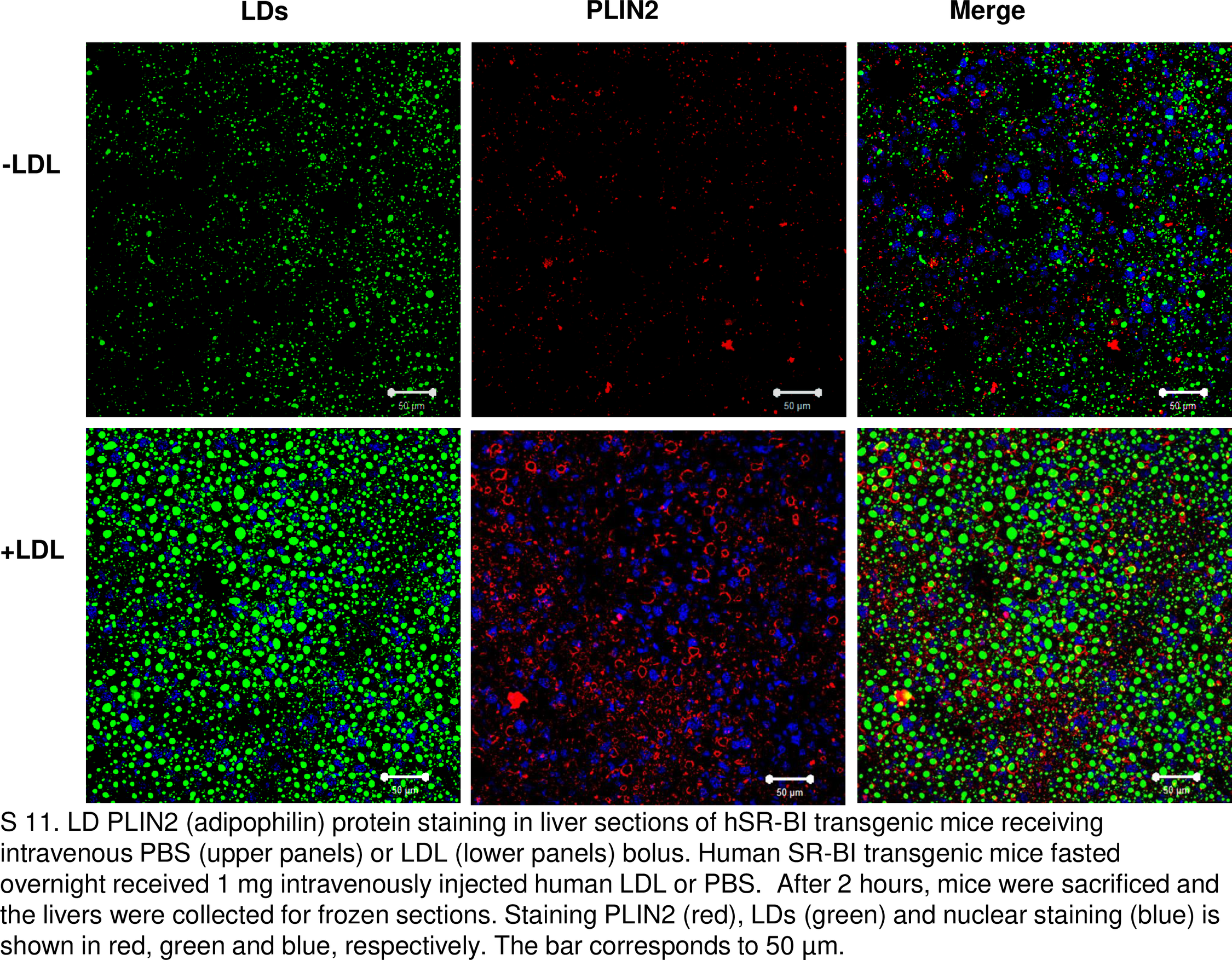

Supplement: S11 Fig — (TIF) [file pone.0240659.s011.tif]

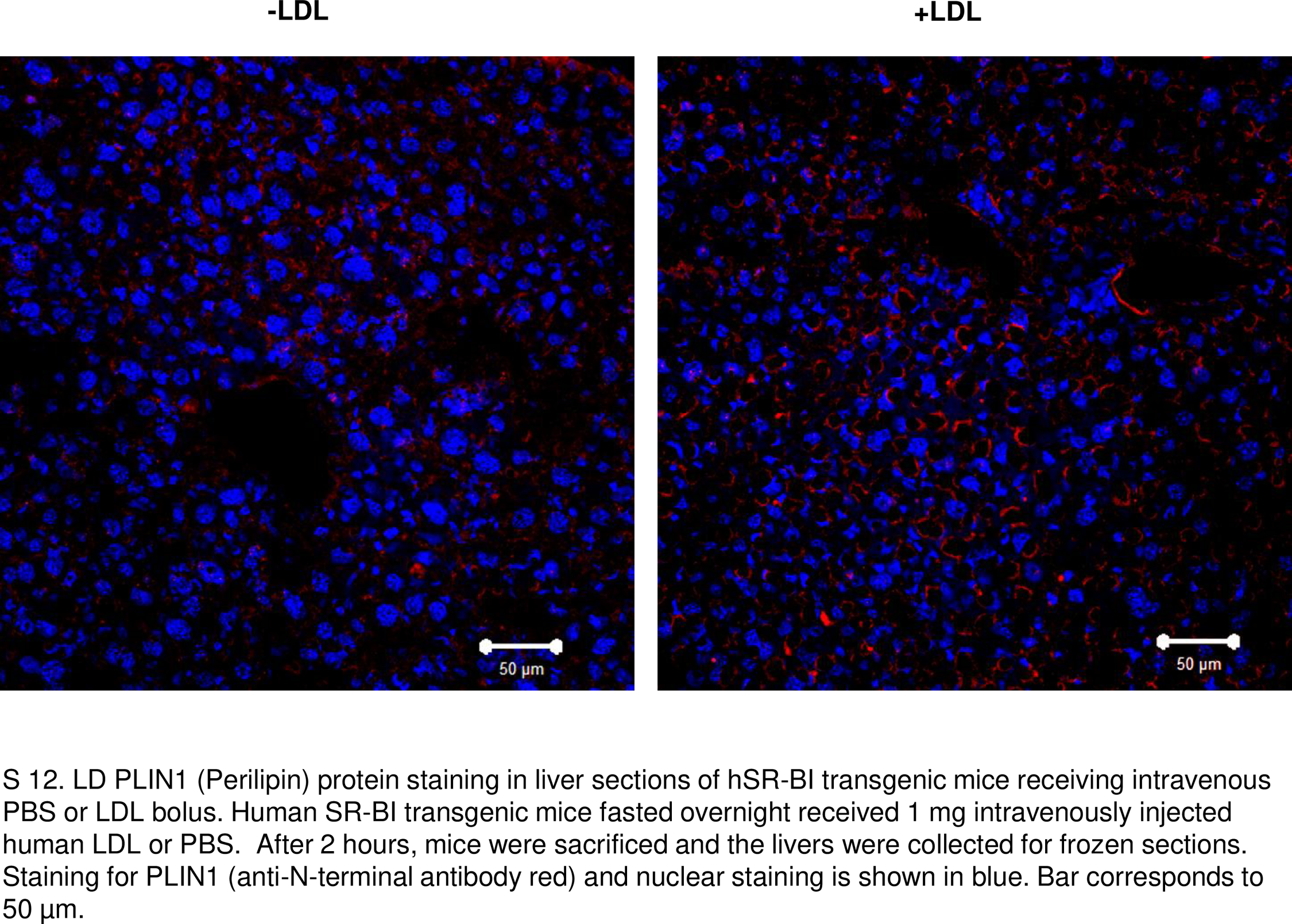

Supplement: S12 Fig — (TIF) [file pone.0240659.s012.tif]
