# Supplementary material for: SR-BI mediates neutral lipid sorting from LDL to lipid droplets and facilitates their formation
Source: PLoS One. 2020 Oct 15;15(10):e0240659. doi: 10.1371/journal.pone.0240659 (PMC7561250; doi:10.1371/journal.pone.0240659)
Supplement: S1 Raw images — (PDF) [file pone.0240659.s013.pdf]

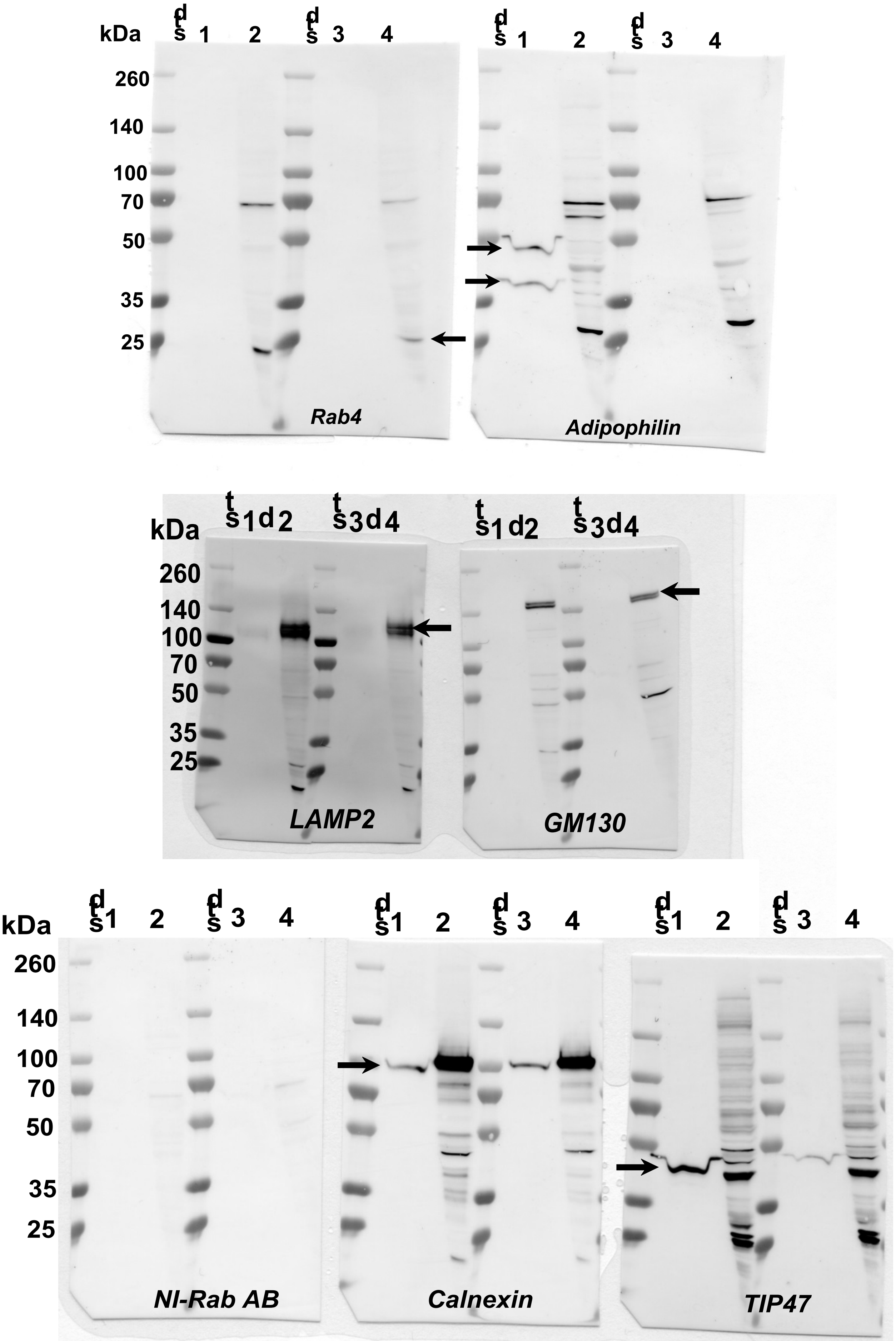

Analysis of the purity of LDs isolated from hSR-BI- and LDLr-expressing HeLa cells

Analysis of the purity of LDs isolated from hSR-BI- and LDLr-expressing HeLa cells. Equal aliquots of LDs isolated from the same amount of SR-BI (lane 1) and LDLr (lane 3) HeLa cells were separated by SDS-PAGE, transferred to NC membranes and immunoblotted with antibodies for adipophilin (LDs), TIP47 (LDs), Rab4 (early endosomes), Calnexin (ER), LAMP2 (late endosomes, lysosomes) and GM130 (Golgi). Expression of the same set of intracellular markers was analyzed using equal percentage of PNS (postnuclear supernatant) from SR-BI (lane 2) and LDLr (lane 4) Hela cells. As control, the same set of samples was incubated with a non-immune antibody of the same isotype (NI-Rab AB) and at the same concentration as the primary antibody, followed by incubation with secondary antibodies and detection reagents.
Arrows indicate the position of the specific bands corresponding to the protein of interest in each blot.

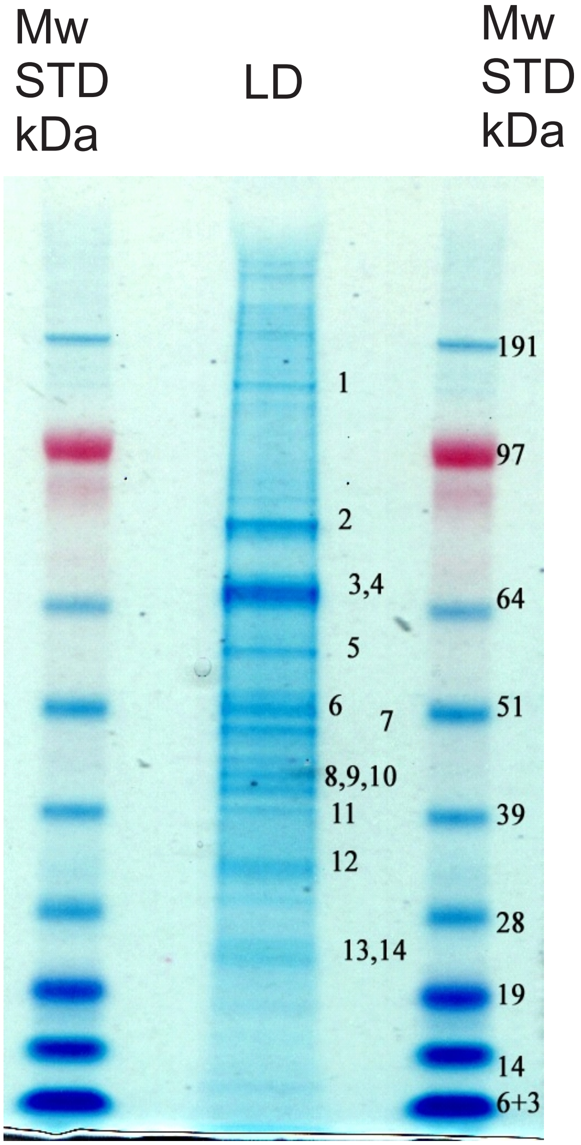

Mass spectrometry of LDs isolated from LD isolates were subjected to SDS-PAGE under reducing conditions.
The bands were cut from the gel as indicated and subjected to MS analysis as described in the Methods and materials section. Lanes 13 and 14 demonstrated no reliable results. Standard bottom proteins Mw of 3 and 6kDa were not separated at these conditions.

Legend for Mass Spectrometry

Sample	*Gene Product*
Sample 1	Collagen type VII
Sample 2	Cytosolic phospholipase A2, group IVA<br>Transferrin receptor variant
Sample 3	Long chain Acyl-CoA synthetase 3 (ACSL-3)
Sample 4	Heat shock 70kDa protein 8 isoform 1 (HSP70)<br>Long chain Acyl-CoA synthetase 3 (ACSL-3)
Sample 5	Chaperonin (HSP60)
Sample 6	ATP synthase beta subunit
Sample 7	Cargo protein TIP47 (PLIN3)
Sample 8	CGI-49
Sample 9	Actin, beta
Sample 10	NAD(P) dependent steroid dehydrogenase-like
Sample 11	Glyceraldehyde-3-phosphate dehydrogenase/<br>aging-associated gene 9 protein
Sample 12	Estradiol 17-beta-dehydrogenase 11/<br>dehydrogenase/reductase (SDR family)
